# Emergence and genomic diversification of a virulent serogroup W:ST-2881(CC175) *Neisseria meningitidis* clone in the African meningitis belt

**DOI:** 10.1099/mgen.0.000120

**Published:** 2017-06-21

**Authors:** Araceli Lamelas, Julia Hauser, Jean-Pierre Dangy, Abdul-Wahab M. Hamid, Katharina Röltgen, Mohamad R. Abdul Sater, Abraham Hodgson, Ali Sie, Thomas Junghanss, Simon R. Harris, Julian Parkhill, Stephen D. Bentley, Gerd Pluschke

**Affiliations:** ^1^​ Swiss Tropical and Public Health Institute, Socinstrasse 57, 4051 Basel, Switzerland; ^2^​ Red de Estudios Moleculares Avanzados, Instituto de Ecologia, Veracruz, Mexico; ^3^​ University of Basel, Basel, Switzerland; ^4^​ Navrongo Health Research Centre, Navrongo, Ghana; ^5^​ Center de Recherche en Sante de Nouna, Nouna, Burkina Faso; ^6^​ Section of Clinical Tropical Medicine, University Hospital Heidelberg, Heidelberg, Germany; ^7^​ Wellcome Trust Sanger Institute, Wellcome Trust Genome Campus, Hinxton, Cambridge CB10 1SA, UK

**Keywords:** *Neisseria meningitidis* serogroup W ST-2881, African meningitis belt, homologous recombination, whole genome sequencing, emerging hypervirulent lineages

## Abstract

Countries of the African ‘meningitis belt’ are susceptible to meningococcal meningitis outbreaks. While in the past major epidemics have been primarily caused by serogroup A meningococci, W strains are currently responsible for most of the cases. After an epidemic in Mecca in 2000, W:ST-11 strains have caused many outbreaks worldwide. An unrelated W:ST-2881 clone was described for the first time in 2002, with the first meningitis cases caused by these bacteria reported in 2003. Here we describe results of a comparative whole-genome analysis of 74 W:ST-2881 strains isolated within the framework of two longitudinal colonization and disease studies conducted in Ghana and Burkina Faso. Genomic data indicate that the W:ST-2881 clone has emerged from Y:ST-175(CC175) bacteria by capsule switching. The circulating W:ST-2881 populations were composed of a variety of closely related but distinct genomic variants with no systematic differences between colonization and disease isolates. Two distinct and geographically clustered phylogenetic clonal variants were identified in Burkina Faso and a third in Ghana. On the basis of the presence or absence of 17 recombination fragments, the Ghanaian variant could be differentiated into five clusters. All 25 Ghanaian disease isolates clustered together with 23 out of 40 Ghanaian isolates associated with carriage within one cluster, indicating that W:ST-2881 clusters differ in virulence. More than half of the genes affected by horizontal gene transfer encoded proteins of the ‘cell envelope’ and the ‘transport/binding protein’ categories, which indicates that exchange of non-capsular antigens plays an important role in immune evasion.

## Abbreviations

CC, clonal complex; GTR, General Time Reversible; HGT, horizontal gene transfer; KND, Kassena-Nankana District; NDSS, Nouna demographic surveillance system; NG, Non groupable; NHD, Nouna Health District; ST, sequence type.

## Data Summary

1. Table S1 and Figs S1−S8 (available in the online Supplementary Material), have been deposited in FigShare: DOI: 10.6084/m9.figshare.4648615.

2. Raw sequencing data for all study genomes have been deposited in the European Nucleotide Archive (ENA). Accession numbers are detailed in Table S1, which has been deposited in FigShare: 10.6084/m9.figshare.4648615.

3. All assembled genomes are available in the NCBI database: (www.ncbi.nlm.nih.gov/nuccore/).

## Impact Statement

Since the early 20th century meningococcal meningitis outbreaks have threatened millions of inhabitants of the African meningitis belt. While in the past serogroup A strains were responsible for most epidemics, serogroup W strains are currently dominating. A first W:ST-11(CC11) epidemic was reported in 2000 among Hajj pilgrims and since then numerous outbreaks related to W:ST-11 strains have occurred. The W:ST-11 clone seems to have emerged through capsule switching from virulent C meningococci and previously W strains had been found to only cause sporadic cases. Surprisingly, an unrelated virulent W lineage associated with ST-2881 emerged in the meningitis belt shortly after the W:ST-11 Hajj outbreak. The W:ST-2881 bacteria belong to clonal complex 175, comprising hypervirulent Y strains that have recently caused outbreaks in the USA, Israel and South Africa. W:ST-2881(CC175) bacteria seem to have emerged through capsule switching from Y to W. While substantial W:ST-2881 outbreaks have occurred in Ghana and Cameroon, the total number of W:ST-2881 meningitis cases seems to be still relatively small. The results of our comparative whole-genome sequencing analysis indicate that horizontal gene transfer events affecting preferentially cell-surface-associated genes have led to the development of several W:ST-2881 clusters with different virulences and epidemic potentials. The results demonstrated that whole-genome sequencing improves discrimination among meningococcal strains, allowing the emergence of new hypervirulent clones to be identified and advancing our understanding of pathogen evolution and spread. The resulting improvement of our understanding and monitoring of outbreak risks can enhance public health measures.

## Introduction

*Neisseria meningitidis* is an obligate commensal, which has adapted to colonize the upper respiratory tract of humans as its only ecological niche. Transmission requires close contact with a colonized person [1]. In colonized individuals meningococci have the potential to cross the epithelial barrier and to enter the bloodstream, where strains belonging to a limited number of hypervirulent lineages may multiply and cause invasive disease, such as sepsis and meningitis [2]. Factors that predispose individuals to invasive meningococcal disease include the lack of bactericidal antibodies and certain complement deficiencies [3]. The polysaccharide capsule covering the cell surface is the most important meningococcal virulence factor and the target for polysaccharide vaccines [3]. Of the 12 serogroups that can be distinguished based on the chemical composition of the capsule, serogroups A, B, C, W, X and Y are responsible for almost all cases of invasive meningococcal disease worldwide. Apart from the capsule, a large number of other genomic traits seem to influence fitness of meningococcal lineages to colonize a human population and to cause invasive disease. This includes proteins involved in adhesion to epithelial cells of the nasopharynx, such as the PilE and PilV components of Type IV pili, the opacity proteins, Opa, and Opc, NadA (Neisseria adhesin A), NhhA (Neisseria hia/hsf homologue) and App (adhesion and penetration protein) [4].

The genomic diversity of *N. meningitidis* is extensive and can be primarily attributed to the natural competence of these bacteria for transformation and homologous recombination. Co-colonization of meningococci with other meningococci and other commensal species of the genus *Neisseria* are the primary source of DNA for horizontal gene transfer (HGT) which leads to shared alleles among genetically distant strains. While HGT results in a highly dynamic population structure, isolates can still be readily grouped into clonal complexes (CC) by the standard seven-gene multilocus sequence typing (MLST) [5]. Sequence types (STs) are grouped into CC by their similarity to a central allelic profile. In the *Neisseria* database a CC includes all STs that match the central genotype at four or more loci. Additional sequence analysis of highly variable genes, such as *porA*, *porB*, *fetA* and *fhbp*, which encode proteins under positive selective pressure [6], and, in particular, comparative whole-genome sequence analysis, can reveal genomic diversity among outbreak strain isolates. An epidemic clonality model has been proposed, which postulates that meningococcal populations undergo occasional rounds of clonal propagation in an otherwise recombining population structure [7]. More recently it has been argued that a predominantly clonal evolution model fits best to the population genetics of *N. meningitidis* [8].

Most meningococcal invasive disease is caused by a limited number of hyperinvasive CC. However, not all strains belonging to a hyperinvasive CC have a similar phenotype and virulence, since the virulence gene content and antigenic makeup may be modified by HGT [9, 10]. Due to the high frequency of recombination, clonal meningococcal populations may differ in recombination blocks acquired through HGT (mostly from other meningococci) and, to a lesser extent, by single-nucleotide polymorphisms (SNPs) caused by point mutations even during rapid spread in the course of an epidemic [10]. Out of the immensely diverse repertoire of genomic variants present in a spreading population, new highly successful ‘fit genotypes’ [11] or immune escape variants [10] may occasionally emerge. The emerging ‘original clone', may rapidly diversify into distinct and recognisable clonal variants during its geographical spread [11].

While incidence rates of invasive meningococcal disease of 0.5–10 cases per 100 000 and per year have been reported for many regions worldwide, rates may be as high as 100–1000 per 100 000 during periodic epidemics occurring in the ‘meningitis belt’ of sub-Saharan Africa [12]. Historically, most of the large epidemics have been caused by serogroup A meningococci. Therefore, vaccination of the population of the meningitis belt with the meningococcal serogroup A capsule glycoconjugate vaccine MenAfriVac was initiated in 2010 [13]. Currently, meningococcal strains expressing the W, but also the C or X polysaccharide, represent the main aetiological agents of bacterial meningitis outbreaks in the meningitis belt [3].

After the first description of *N. meningitidis* serogroup W in 1968 [14] sporadic cases of severe invasive disease caused by W meningococci have been identified in Northern Europe and the USA [15]. In 2000, a first large outbreak caused by W meningococci was reported among Hajj pilgrims in Mecca [16]. Since then epidemics caused by W:ST-11(CC11) meningococci have occurred in the African meningitis belt and outbreaks have been reported worldwide. Results of whole-genome sequencing analyses have indicated that all W:ST-11 isolates were likely to be descendants of a single ancestral strain [17]. Hyperinvasive meningococcal isolates belonging to CC11 express C, W, or less frequently B or Y capsule polysaccharide. The genomes of CC11 strains form a single main lineage comprising two sublineages, 11.1 and 11.2 [18]. Serogroup W:ST-11 isolates are confined within sublineage 11.1, forming several clusters in addition to the ‘original’ Hajj outbreak clone. It is assumed that the W:ST-11 clone has emerged in recent times through capsule switching. Results of analyses of the capsule synthesis gene cluster indicate that the capsular switch involved two separate recombination events and that the vast majority of W CC11 strains have retained both recombination blocks [19]. The Hajj clone has undergone additional recombination events involving the acquisition of virulence genes. Currently, appreciable amounts of invasive meningococcal disease cases worldwide are caused by W CC11 strains which are distinct from the Hajj clone. The distinct genotypes can only be identified by whole-genome sequencing analysis, and appear to translate into distinct phenotypes which can exhibit different clinical manifestations, such as a higher case fatality rate of infections caused by the ‘South American/UK’ W CC11 strains, in comparison to the original Hajj clone [18, 19].

A highly virulent W meningococcal lineage unrelated to the CC11 meningococci and associated with ST-2881 belonging to CC175 [20] was first described in Niger, where it emerged in 2002 and caused in 2003 sporadic meningitis cases in Niamey [21, 22] and a cluster of cases in the Illela district [23]. In a carriage study in the Illela district, ST-2881 strains with different capsule polysaccharides (W and Y), but the same pulsed-field gel electrophoresis pattern and (NT):P1.5,2 phenotype were isolated, indicating that the W:ST-2881 clone emerged from Y:ST-2881(CC175) bacteria by capsule switching [23]. In 2003 W:ST-2881 strains were also isolated in Benin, Nigeria [22] and Burkina Faso [24]. While W:ST-2881 meningococci represented in 2007 and 2008 the vast majority of meningococcal meningitis case isolates in Cameroon, since 2009 W:ST-11(CC11) strains have predominated [25]. Within the framework of two longitudinal colonization and disease studies [26, 27] we have monitored outbreaks caused by W:ST-2881 meningococci in the Kassena-Nankana District (KND) of Ghana in 2009–2011 and in the Nouna Health District (NHD) of Burkina Faso in 2005–2009. Results of comparative whole-genome analyses reported here demonstrate rapid diversification of the W:ST-2881 clone and provide evidence for differences in virulence and epidemic potential of different W:ST-2881 clusters.

## Methods

### Study sites

The serogroup W *N. meningitidis* isolates investigated in this study have been collected in the KND of Ghana and in the NHD of Burkina Faso. Case strains were isolated from the cerebrospinal fluid (CSF) of meningitis patients, and carriage strains were isolated from throat swabs collected during longitudinal carriage surveys. Isolation and characterization of bacterial isolates has been described previously [26, 28, 29].

The NHD lies in the Kossi region of Burkina Faso. The Nouna Health and Demographic Surveillance System (NounaDSS) covers a part of the NHD with a current population size of about 99 000 people. The NounaDSS is distributed into 58 villages and Nouna city. The population of the district is mainly rural, excluding the inhabitants of Nouna city [27]. The NHD has dry savannah vegetation with a long dry season lasting from October to May and a short rainy season during the rest of the year. For a longitudinal meningococcal colonisation study with a sample size of about 300 participants, 37 compounds were selected by proportional cluster sampling from the NounaDSS population. A second longitudinal study was initiated in a village located close to Ira outside the NounaDSS area after a focal serogroup A meningococcal meningitis outbreak in 2006 [29]. The KND lies in the Upper-East Region of Ghana within the Guinea savannah woodland and has two major seasons: a short wet season from June to October and a long dry season for the rest of the year. The district population is about 140 000, mainly rural, except for the 20 000 inhabitants of Navrongo town. In the KND, people live in compounds with an average of ten inhabitants. For the colonization study with a sample size of about 300 participants, 37 residential compounds from the complete listing of the district population were selected using the Navrongo Demographic Surveillance System (NavrongoDSS) [26]. In all three colonization studies throat swabs were taken twice annually from all consenting members of the selected compounds present at the time of the visit. All isolates included in the whole genome sequencing study (Table S1) came from different persons.

### Ethical clearance

Ethical clearance was obtained by the institutional and national ethical committees in Burkina Faso and Ghana and from the ethical committee of the University Hospital Heidelberg.

### Preparation and sequencing of genomic DNA

*N. meningitidis* strains (Table S1) were grown in liquid Brain–Heart Infusion (Bacto) medium and chromosomal DNA was prepared as described previously [30, 31]. Briefly, bacterial pellets were resuspended in 0.5 ml TES buffer [50 mM Tris, 20 mM EDTA, 50 mM NaCl (pH 8)]. A 2 µl volume of RNase A (Qiagen 100 mg ml^−1^) and 20 % SDS were added to a final concentration of 1 % and cells were lysed for 5 min at 42 °C. After two phenol/chloroform/isoamyl alcohol (25 : 24 : 1, Sigma) extractions and one chloroform/isoamyl alcohol (24 : 1, Sigma) extraction, DNA was precipitated in two volumes of isopropanol followed by suspension in TE buffer [10 mM Tris, 1 mM EDTA (pH 8)]. Ammonium acetate was added to a final concentration of 2.5 M and the DNA was precipitated in two volumes of ethanol. After two 70 % ethanol wash steps and subsequent drying, the DNA was diluted in TE buffer [10 mM Tris, 0.1 mM EDTA (pH 8)].

Multiplexed genomic DNA libraries were prepared with an insert size of 200 using 24 unique index tags. Libraries were combined into pools of 24 and sequenced on an Illumina HiSeq for 75 cycles from each end to produce paired-end reads plus an eight-base index sequence read. Downstream analysis used the index tags to assign reads to individual samples.

### Read alignment and SNP detection

To provide a high-quality reference sequence, the genome of the W:ST-2881 strain 2039 (Table S1) was sequenced using single-molecule, real-time (SMRT) technology on a PacBio RS II (Pacific Biosciences) with the P5-C3 chemistry. One SMRT cell was used with a movie length of 180 min. Strain 2039 was isolated in 2005 in Burkina Faso and was selected as a reference strain because it was the oldest isolate among our W ST-2881 strain collection. The genome was assembled *de novo* using the hierarchical genome assembler (HGAP in SMRTAnalysis v2.3.0) [32]. The assembly contained two contigs with a large overlap, which were manually joined and trimmed to remove end overlaps caused by the circularity of the chromosome. The sequence was then corrected using Illumina sequence data for the same isolate using RATT [33] and annotated using PROKKA v. 1.11 [34]. Sequence data has been deposited in the ENA under study accession number ERP002590 and sample accession number ERS323054. Comparison and visualization of the strain 2039 genome (Fig. S1) was carried out using BRIG (http://sourceforge.net/projects/brig/) [35].

Variation in the form of single-nucleotide polymorphisms (SNPs) among the sequenced W:ST-2881 isolates (Table S1) was detected using a mapping approach. The paired-end Illumina reads were mapped against the *N. meningitidis* serogroup W:ST-2881 strain 2039 as reference (accession numbers ERR369484) with an insert size of between 50 and 400 bp using SMALT (www.sanger.ac.uk/resources/software/smalt/). SNPs were identified using SAMtools [36] as previously described [37]. SNPs called in phage sequences and repetitive regions of the *N. meningitidis* reference genome were excluded. Repetitive regions were defined as exact repetitive sequences of ≥50 bp in length, identified using rep repeat-match. If 10 % of the genomes had an indetermination in a called SNP, these positions were removed from the analysis.

### Identification of recombination events

Ancestral sequences were reconstructed onto each node of the phylogeny using PAML [38]. From these ancestral sequences, SNPs were reconstructed onto branches of the tree. To identify recombination events, we applied the moving window approach as previously described [39]. The Gubbins (Genealogies Unbiased By recomBinations In Nucleotide Sequences) approach is used to identify recombination blocks using an algorithm that iteratively identifies loci containing elevated densities of base substitutions while concurrently reconstructing a phylogeny based on the putative point mutations outside of these regions. Sequences of all strains with possible recombination fragments were assembled using velvet v1.0.12 [40] *de* *novo* assemblies with contigs realigned by Abacas [41]. Draft genomes were used to query the 2039 genome using blastn, and comparison files were generated and viewed using the Artemis Comparison Tool (ACT) [42]. In order to confirm the recombination fragments and to identify the most closely related alleles, the predicted recombination fragments and their upstream and downstream flanking regions were extracted from the assembled genomes and aligned with their homologues from 15 publicly available genomes of strains of *N. meningitidis*[Z2491 (AL157959.1), WUE2594 (FR774048.1), 2335 (ERS041026), 1264 (ERS040961), M01-240355 (CP002422.1), M01-240149 (CP002421.1), M04-240196 (CP002423.1), NZ05/33 (CP002424.1), alpha710 (CP001561.1), H44/76 (CP002420.1), MC58 (AE002098.2), G2136 (CP002419.1), FAM18 (AM421808.1), 8013 (FM999788.1), ST-53 a14 (NC_013016)], three *N. gonorrhoea* [FA1090 (AE004969.1), TCDC-NG08107 (PRJNA61377) and TCDC-NG08107 (CP002440.1)] and one *N. lactamica* [020-06, (FN995097.1)] genomes. The alignments were used to reconstruct maximum-likelihood phylogenetic trees with RAxML v7.0.4 [43] using a general time-reversible (GTR) substitution model with γ correction for among-site rate variation. Support for nodes on the trees was assessed using 100 bootstrap replicates. We considered a recombination fragment confirmed when the cluster differed from the clusters belonging to the upstream and downstream flanking regions. Recombination hotspots were defined as regions in a genome that exhibit elevated rates of recombination, relative to a neutral expectation [44, 45].

### Phylogenetic analyses

A maximum-likelihood phylogenetic tree was generated from the whole-genome sequence alignment without SNPs associated with recombination events. Phylogenetic trees were reconstructed as described above.

### Relating study isolates to known diversity

To analyse the phylogenetic relationship between our W:ST-2881 reference genome sequence of strain 2039 from Burkina Faso and genome data available in the Meningitis Research Foundation Meningococcus Genome Library (www.meningitis.org/research/genome) for the ST-175 strains M22783, M22804, M22809, M22811, M22819, M22822, M22828, 16570, 677, 8465, 8246, 13160, 11244, 12870, 1026, 9371, 9558, 44133, 9634, 10369, 15734, M15_240953, LNP28435, 16-180, 2007461, PT42, PT56, the ST-167 strains 159000102, 359000020, 159000325, 96062 and the ST-8447 strain 9281, the strains were compared by using the Genome Comparator tool located in the pubMLST database (https://pubmlst.org/neisseria) with the ‘All loci’ option [46]. We calculated a distance matrix between the strains based on the number of loci that differed between each pair of isolates. The Genome Comparator tool conducts a gene-by-gene comparison of isolates. A distance matrix is generated as the result of allelic variation the isolates exhibit at the different loci included in the comparison. The NeighbourNet algorithm [47] resolves the relationship among the isolates into a network of relatedness, visualized in SplitsTree 4 (version 4) [48]. Alleles of the MLST genes and of the *fetA*, *porB*, *Nhba*, *opcA* and *fHbp* genes were determined with the tool ‘Sequence query – Neisseria profile/sequence definitions’ (https://pubmlst.org/neisseria).

## Results

### Outbreaks of W:ST-2881 meningococcal meningitis in Ghana and Burkina Faso

**Fig. 1.**
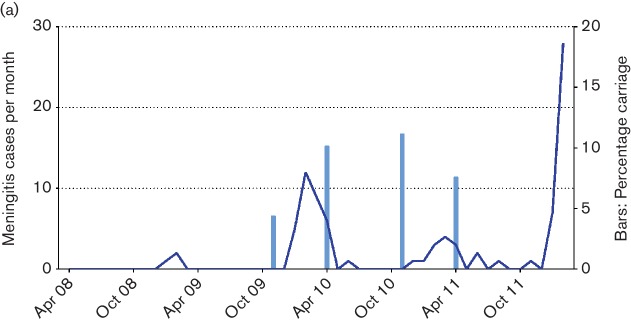
Dynamics of *N. meningitidis* colonisation and meningitis in the KND from November 2007 until February 2012. Carriage rates recorded during colonisation surveys (April and November each year) and monthly numbers of confirmed meningitis cases caused by serogoup W:ST-2881 meningococci.

Within the framework of longitudinal meningococcal disease and colonization studies in the Kassena-Nankana District (KND) of Northern Ghana and the Nouna Health District (NHD) of Burkina Faso [26, 29], we have observed outbreaks of W:ST-2881 meningococcal meningitis. The 74 sequenced W:ST-2881 *N. meningitidis* carried (*n*=48) and disease (*n*=26) isolates described here were collected in the KND from 2009 to 2011 (65 isolates) and in the NHD from 2005 to 2009 (9 isolates) (Table S1). Isolates from the NHD of Burkina Faso came either from the area of the NounaDSS in the south of the NHD or from a health centre located in the village Ira outside the NounaDSS in the north of the NHD [29]. While we observed only sporadic W:ST-2881 carriers and meningitis cases in the NHD, a substantial W:ST-11 outbreak started in 2011 in the NounaDSS area (Gerd Pluschke, unpublished results). In the KND, we observed a wave of W:ST-2881 colonization and disease between 2009 and 2011 with a peak monthly incidence rate of 20 meningitis cases per 100 000 in February 2010 and peak colonization rates of 15.2 and 16.7 % in April and November of 2010, respectively (Fig. 1).

### Whole-genome comparison of W:ST-2881 strains with other meningococci

**Fig. 2.**
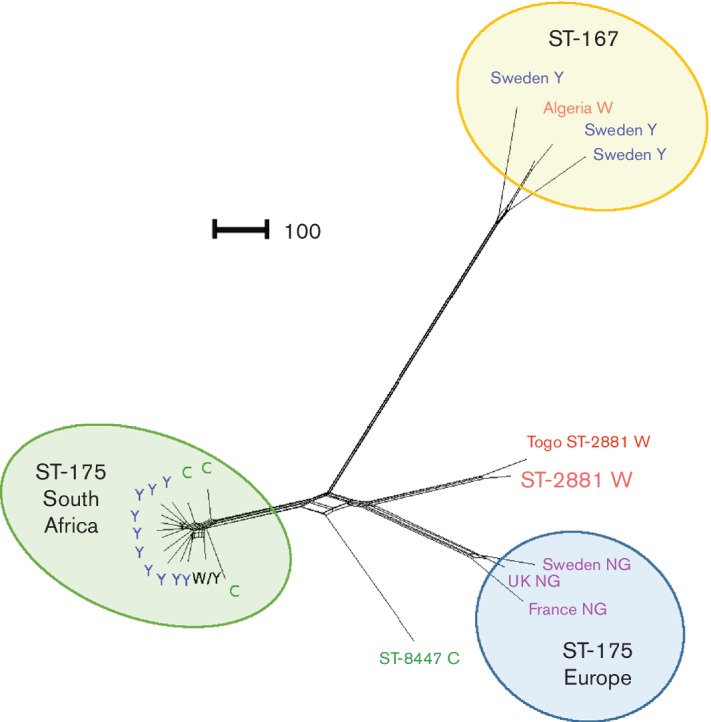
NeighborNet graph generated from the distance matrix calculated by the tool Genome Comparator using the genome of the W:ST-2881 strain 2039 as a reference genome and the available genome sequencing data of the most closely related strains and clonal complexes. The scale bar indicates the number of differences among the 1990 loci compared.

Using the tool Genome Comparator located in the pubMLST database (pubmlst.org/neisseria), we identified *N. meningitidis* strains with available genome data that were most closely related to the W:ST-2881 isolates. Using the genome sequence of the W:ST-2881 strain 2039 from Burkina Faso, for which the genome was analysed using the PacBio sequencing platform, as a reference and published *N. meningitidis* genome sequencing data, we identified strains belonging to CC167 and CC175 (Table 1) as being most closely related. Based on the number of loci that differed between each pair of isolates (allelic differences) a distance matrix was calculated using the Genome Comparator tool, which represents the equivalent of a whole-genome MLST profile. A NeighborNet graph generated from the distance matrix (Fig. 2) showed that the W:ST-2881(CC175) strain 2007461,which was isolated in 2007 in Togo (Table 1), was the most closely related isolate with published genome sequencing data. However, the W:ST-2881 strains from Ghana and from Burkina Faso were more closely related to each other than to this W:ST-2881 strain from Togo (Fig. S2). The most closely related CC was CC175 comprising a cluster of Y and C isolates from South Africa and another cluster of non-groupable ST-175 strains from Europe (Fig. 2). Y and W strains belonging to CC167 were less closely related to the W:ST-2881 strains than CC175 (Fig. 2). Whole-genome analysis-based data corresponded well with standard seven-gene MLST. ST-2881 is a single-locus variant of ST-175, which is associated with the *abcZ* allele 6, instead of the allele 179 present in the ST-2881 strains. ST-167 differs from both ST-2881 and ST-179 at three (*abcZ*, *aroA* and *fumC*) of the seven standard MLST gene loci.

**Table 1.** Allelic profile (*porA, porB, fetA, nba, opcA* and *fhbp* genes) of the W ST-2881 clusters from Ghana and Burkina Faso and of related CC175 and CC167 isolates. Yellow colour: W:ST-2881 cluster; green colour: alleles also present in W:ST-2881 strains. NG, non groupable.

ID	Isolate/cluster	Country	Year	Serogroup	ST	Clonal complex	NEIS1364 (porA)	NEIS1963 (fetA)	NEIS2020 (porB)	*NEIS2109 (nhba)*	NEIS2198 (opcA)	NEIS0349 (fhbp)
	cluster A, B	Burkina Faso	2005–09	W	2881	CC175	675	1009	234	7	3	1
	cluster C4, C5	Ghana	2010	W	2881	CC175	675	1009	234	7	3	1
	cluster C1, C2, C3	Ghana	2009–10	W	2881	CC175	675	1009	234	7	3	774
49369	2007461	Togo	2007	W	2881	CC175	675	1009	234	7	3	1
41526	M15_240953	UK	2015	NG	175	CC175	1046	1009	234	7	3	130
41727	LNP28435	France	2016	NG	175	CC175	1046	1009	234	7	3	598
42784	16-180	Sweden	2016	NG	175	CC175	1046	1009	234	7	3	130
40583	9281	South Africa	2003	C	8447	CC175	202	474	–	7	6	54
40588	9634	South Africa	2003	C	175	CC175	–	474	36	7	3	619
40593	10369	South Africa	2003	C	175	CC175	202	474	36	7	3	84
40644	15734	South Africa	2004	C	175	CC175	–	474	36	7	–	84
40423	44133	South Africa	2014	W/Y	175	CC175	–	474	36	7	3	84
25822	16570	South Africa	2004	Y	175	CC175	202	474	36	Incomplete	3	84
25823	677	South Africa	2005	Y	175	CC175	202	474	36	Incomplete	3	84
25826	8465	South Africa	2006	Y	175	CC175	202	474	36	Incomplete	–	84
25829	8246	South Africa	2006	Y	175	CC175	202	474	36	Incomplete	–	84
25834	13160	South Africa	2004	Y	175	CC175	202	474	36	Incomplete	–	84
25840	11244	South Africa	2007	Y	175	CC175	202	474	36	7	–	84
25847	12870	South Africa	2007	Y	175	CC175	–	474	36	Incomplete	3	84
25852	1026	South Africa	2005	Y	175	CC175	996	474	36	Incomplete	3	84
25872	9371	South Africa	2003	Y	175	CC175	202	–	36	7	–	84
25874	9558	South Africa	2003	Y	175	CC175	202	474	36	7	–	84
30102	96062	Algeria	1996	W	167	CC167	63	993	29	7	3	4
26096	159000102	Sweden	2001	Y	167	CC167	63	5	42	7	3	4
26102	159000325	Sweden	2001	Y	167	CC167	63	504	29	7	3	203
26108	359000020	Sweden	2003	Y	167	CC167	127	1748	29	*7*	3	4

### Phylogenetic analysis of serogroup W:ST-2881 *N. meningitidis* isolates

To gain insight into the phylogenetic relationships between the W:ST-2881 isolates we performed a genome-wide SNP analysis. The obtained Illumina sequences (average coverage 319 reads per position) were mapped against the genome of the W:ST-2881 strain 2039 from Burkina Faso. Our sequences covered 85.2 % of the complete 2039 reference genome. SNP calling revealed a total of 1615 SNPs within the non-repetitive genome (1 867 404 bp). SNPs associated with recombination blocks were removed as described previously [39] and a maximum-likelihood phylogenetic tree was reconstructed using a General Time Reversible (GTR) evolutionary model based on the 145 SNPs not associated with recombination blocks. Hence, 91.1 % (1470/1615) of all called SNPs were associated with recombination blocks and only 8.9 % of them thus represent putative point mutations.

**Fig. 3.**
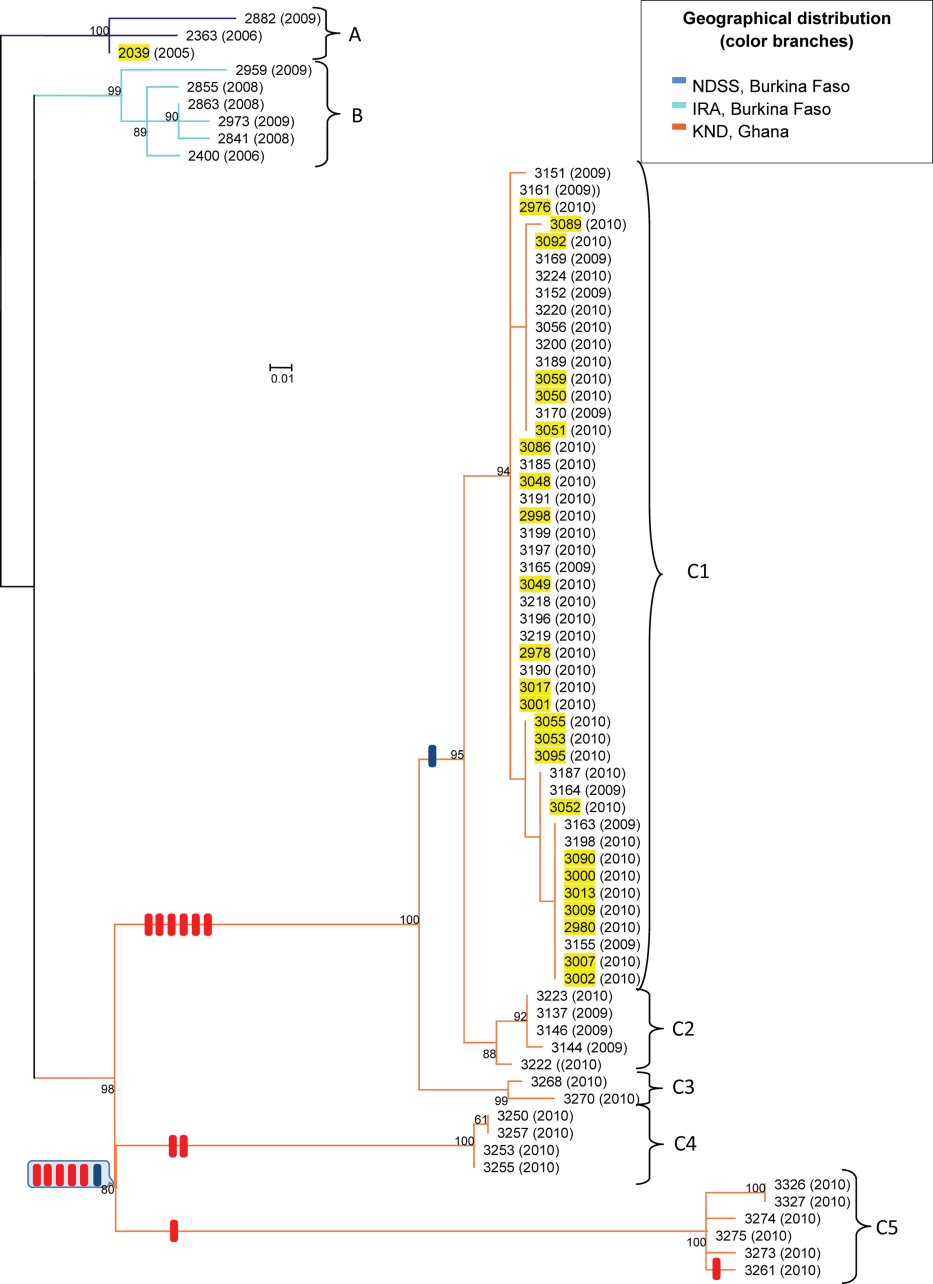
Unrooted maximum-likelihood phylogenetic tree based on 145 putative point mutations, excluding predicted recombination events. Branches are coloured on the basis of the region of isolation of the strains. Recombination events are indicated along the branches by coloured boxes. The blue boxes correspond to recombination events in the *yggS*–*pilT*–*pilU* locus. Strains isolated from the CSF of meningitis patients are marked with yellow. Bootstrap values above 50 % are indicated along the branches.

The generated phylogenetic tree reflects a highly structured clonal population composed of three closely related phylogenetic variants associated with the geographic origin of the strains (Fig. 3). The strains from the NHD of Burkina Faso formed two separate variants (designated A and B in Fig. 3) with variant A containing all three strains from the NDSS area in the south of the district and variant B containing all six strains from the Ira Health Centre in the north of the NHD. Variant C, formed by the 65 strains from the KND of Ghana, was divided into five clusters, designated C1–C5 (Fig. 3). There was no association of the clusters with the year of isolation, but remarkably, all 25 Ghanaian disease isolates clustered together with 23 of the Ghanaian isolates associated with carriage in cluster C1. The remaining 17 carried isolates were distributed over clusters C2–C5.

Within the analysed set of W:ST-2881 strains, the highly variable *porA*, *porB*, *fetA*, *nba*, *opcA* and *fhbp* genes, which are often used as additional molecular epidemiological markers, showed only very limited diversity (Table 1). The isolates from Ghana and Burkina Faso harboured the same *porA*, *porB*, *fetA*, *nhba* and *opcA* alleles as the W:ST-2881 isolate 2007461 from Togo [(NEIS1364 (*porA*): 675; NEIS2020 (*porB*): 234; NEIS1963 (*fetA*): 1009; NEIS2109 (*nhba*): 7; NEIS2198 (*opcA*): 3]. However, diversity was found in *fhbP*, which is related to a recombination event involving the *fhbP* gene (Fig. S7). While the *fhbP* allele 1 present in the Togo isolate was also found both in all isolates from Burkina Faso and in all cluster C4 and C5 strains from Ghana, Ghanaian isolates belonging to the clusters C1, C2 and C3, harboured the allele 774. Among the CC167 and CC175 strains, the non-groupable ST-175 isolates from Europe (Fig. 3) had the most similar allelic profile, with four out of six identical alleles (Table 1). Only one point mutation was found in the six genes: strain 2959 has a non-synonymous SNP at position 578 (A→C) producing an amino acid exchange (Q→ P) in the *porA* gene.

### Analysis of the phylogenetic history of recombination blocks

To elucidate the role of recombination events in the genomic diversification of the W:ST-2881 meningococci, we investigated their recombination history. A total of 17 recombination blocks with high SNP density were predicted by the method of Croucher *et al.* [39] and confirmed by phylogenetic analysis (Figs S3–S18). The size of the identified recombination blocks, which are spread over the genome (Fig. S1), varied between 312 and 9323 bp, with a mean length of 3460 bp (Table 2), affecting altogether 2.7 % of the total genome length of 2 185 600 bp.

**Table 2.** Properties of the 17 recombination blocks identified in the genomes of the W ST-2881 strains

Recombination fragment	Length (bp)	Number of SNPs	Present in cluster/strain	Genes located in the recombination fragments	Phylogenetically closest species/strain	Figure
28 6084−288 299	2216	156 (9 Int/31 N/116 S)	3261	*yfhA*, *feuB*, *fetB*	*N. meningitidis* (MC58)	Fig. S17
49 885−52 503	2619	98 (13 Int/37 N/48 S)	C1-C2	*yggS*, *pilT*, *pilU*	*N. lactamica*	Fig. S13
238 848−239 768	921	24 (2 Int/5 N/17 S)	C1-C2-C3	*ybeB*, *rmlH*	*N. meningitidis* (2335, 1264, WUE2594)	Fig. S2
501 192−503 139	1948	9 (1 STOP/3 N/5 S)	C1-C2-C3	*pilP*, *pilO*, *pilM*, *pilN*	*N. meningitidis* (alpha710, M04-240355)	Fig. S3
681 556−681 867	312	17 (9 Int/5 N/3 S)	C1-C2-C3	NM2039_00671	*N. meningitidis*	Fig. S4
1 755 144−1 756 161	1018	50 (17 N/33 S)	C1-C2-C3	*abgT*	*N. meningitidis* (2335, 1264, WUE2594)	Fig. S5
1 808 944−1 813 086	4143	195 (2 STOPs/4 Int/104 N/85 S)	C1-C2-C3	*tbpA*, *Tbp2*	*N. lactamica*	Fig. S6
421 704−422 869	1166	78 (10 Int/9 N/59 S)	C1-C2-C3	*fda*, *fhbP*	*N. meningitidis* (MC58, H44-76)	Fig. S7
64 227−64 909	683	13 (1 N/12 S)	C4	*ftsY*	*N. meningitidis* (8013)	Fig. S14
362 486−370 050	7565	249 (49 Int/48 N/152 S))	C4	*glyS*, *lgtA*, *Lex1*, NM2039_00355, *lgtE*, *rsmE*, *suhB_1*	*N. gonorrhoeae*	Fig. S15
662 403−663 594	1192	108 (1 STOP/11 N/96 S)	C4-C5	*ppsA*	*N. lactamica*	Fig. S11
934 662−943 455	8794	109 (9 Int/27 N/73S)	C4-C5	*lst*, NM2039_00941, *ydfG*, NM2039_00943, NM2039_00944, *piiC_1*, *pip*, NM2039_00947, *dapA*	*N. meningitidis*	Fig. S12
47 875−54 519	6645	111 (5 Int/34 N/72 S)	C4-C5	*dksA*, proC, NM2039_00045*, yggS*, *pilT*, *pilU*, *yccS*	*N. meningitidis* (2335, 1264, WUE2594)	Fig. S13
125 372−132 608	7237	47 (15 Int/7 N/25 S)	C4-C5	*rssA1, prfB, dtpT*, NM2039_00122	*N. meningitidis* (8013)	Fig. S8
39 406−41 371	1966	38 (3 Int/4 N/31 S)	C4-C5	NM2039_00037, *DnaJ_1*	*N. meningitidis* (8013)	Fig. S9
333 713−343 035	9323	75 (6 Int/16 N/53 S)	C4-C5	*appY*, NM2039_00318, *mlaE*, *mlaD*, *mlaC*, NM2039_00322, *mlaA*, NM2039_00324, NM2039_00325, NM2039_00326, NM2039_00327, *rpmE*, NM2039_00329, NM2039_00330	*N. meningitidis*	Fig. S10
455 674−456 745	1072	56 (4 Int/10 N/42 S)	C5	NM2039_00443, *fhs*	*N. meningitidis* (8013)	Fig. S16

Int, intergenic SNP; N, non-synonymous; S, synonymous; STOP, STOP codon.

The locations of the recombination events, which were all associated with the genomic diversification within cluster C, across the phylogenetic tree, are depicted in Fig. 3. Strains belonging to clusters C1, C2 and C3 have six recombination blocks in common, and all strains of clusters C1 and C2 shared one additional recombination block. Also, all C4 and C5 strains have six recombination blocks in common; all C4 strains shared two and all C5 strains one additional recombination block. Furthermore, one block was present in only a single C5 strain (3261). In order to elucidate the putative origins of the recombination blocks, we performed an *a posteriori* phylogenetic analysis (Figs S3–S18). The most closely related genes were found in one case in *N. gonorrhoeae*, in two cases in *N. lactamica* and in the other 14 cases in strains of *N. meningitidis* (Table 2).

The 17 recombination blocks comprised altogether 61 genes (Figs S3–S18). Functional classification of the genes affected by the recombination events revealed that genes encoding proteins of the ‘cell envelope’ (20/61) and the ‘transport/binding protein’ (12/61) categories were over-represented, since 32.8 and 19.7 % of the affected genes belonged to these two categories, while these comprised only 16.3 and 6.3 % of all genes, respectively [49]. One genomic region, the *yggS–pilT–pilU* locus was associated with two independent recombination events (Fig. S13). While a 6645 bp fragment spanning the genomic region from *dskA* to *yccS* was common to all C4 and C5 strains, a smaller (2619 bp) fragment was common for all C1 and C2 strains (Fig. 3). This locus encodes the PilU and PilT proteins, which are involved in the regulation of type IV pilus expression and has been identified before as recombination hotspot in a comparative whole-genome analysis of 100 serogroup A strains [10].

## Discussion

The only habitat of *N. meningitidis* is the human nasopharynx and many meningococcal lineages have evolved that compete for colonization of this niche. In meningococcal carriage studies in Europe diverse and relatively temporally stable colonizing meningococcal populations have been found that primarily consist of strains belonging to CC that generally exhibit low virulence [50, 51]. In contrast, in the African meningitis belt, clonal waves are a characteristic feature of meningococcal colonization [26] and outbreaks of invasive disease occur, when the dominating colonizing population belongs to a hypervirulent clone. The genomic basis of hyperinvasiveness of certain clones is not clear, a core pathogenome has not been identified and comparative whole-genome sequencing studies, including the present analysis, have not revealed systematic differences between clonally related disease and carriage isolates [10].

Phylogenetic studies have revealed that meningococcal populations are structured as consequence of clonal decent, HGT and point mutations. In particular HGT leads to the development of an immensely diverse repertoire of genomic variants during clonal spread and new ‘fit genotypes’ [11] may emerge occasionally. Their spread may remain unnoticed, if they do not have hypervirulent properties. Recent examples for the emergence of new hypervirulent clones are W:ST-11(CC11) meningococci, which appear to have evolved from hypervirulent CC11 C meningococci by capsule switching [19] and A:ST-2859 meningococci, which have evolved from the hypervirulent A:ST-7 clone and successfully spread after the accumulation of 13 recombination blocks, leading to immune evasion through complex changes in cell surface structures of the emerging clone [10]. The W:ST-2881 meningococci described here represent another example of a recently emerged and successful meningococcal clone. In a carriage study in Niger, closely related ST-2881 strains expressing either the W or the Y capsule have been isolated, indicating that Y:ST-175 strains may have first developed into Y:ST-2881 strains and then switched the capsule, resulting in the new highly successful W:ST-2881 clone. The W and Y capsular polysaccharides are closely related and composed of alternating sequences of d-galactose or d-glucose and sialic acid [52]. While the enzymes encoded by *cssA–cssC* that synthesize CMP-*N*-acetylneuraminic acid are conserved among serogroups Y and W, the sialyltransferases encoded by the *csw* and *csy* genes, which catalyse the transfer of sialic acid to galactose or glucose, respectively [53], are serogroup-specific. Due to the similarity of the region A of the *cps* locus, we were not able to dissect the recombination event(s) associated with the Y to W capsule switch in the progenitor of the W:ST-2881 bacteria. CC175 strains with the Y capsular polysaccharide were described for the first time in 1999 in South Africa [54] and apart from the Y and W isolates, B and C strains belonging to this CC have also been isolated (pubmlst.org).

During the spread of a clonal epidemic, individual examples of the clone can exhibit micro variation due to HGT and point mutations. The ‘original clone’ can thus diversify into distinct and recognisable variants, as it spreads over time. Variants may inhabit different niches of the nasopharyngeal ecosystem, as has been indicated for pneumococcal lineages [55]. These variants may exhibit differences in genes encoding proteins, which may alter the strain’s capacity to cause invasive meningococcal disease. It has been suggested that W:ST-2881 strains have a lower virulence than W:ST-11 strains and that extensive circulation and asymptomatic carriage of W:ST-2881 strains may have prevented an epidemic of the potentially more virulent W:ST-11 lineage in Niger [56]. In fact, in spite of their widespread occurrence in the meningitis belt, the total number of meningitis cases caused by the W:ST-2881 clone seems to be much more limited than those caused by W:ST-11 meningococci. On the other hand, substantial W:ST-2881 outbreaks have been recorded in Cameroon and in Ghana. The phylogenetic analysis presented here has revealed a highly structured clonal population with several distinct clusters. The fact that all Ghanaian disease isolates clustered with corresponding carriage isolates in one of the five variant C clusters is indicative of different virulence capacities within the W:ST-2881 population. This underscores the necessity of comprehensive and continuous strain characterization using whole-genome sequencing, to distinguish the minor genotypic differences among circulating strains which demonstrated differential capacities to cause invasive meningococcal disease. It remains to be seen whether certain W:ST-2881 variants will persist as colonizers in the population of the African meningitis belt and whether these have epidemic potential.

Based both on the analysis of point mutations and the presence of recombination blocks, the W:ST-2881 variant C, which colonized the population of the KND in 2009/10, could be differentiated into five clusters. Clusters C1, C2 and C3 had six recombination blocks in common and the two clusters C4 and C5 had another six blocks in common. In addition, five more recombination events were identified among the 65 variant C isolates. Similar to results describing clonal serogroup A meningococcal isolates [10], the *pilT–pilU* locus, regulating type IV pilus expression, was identified as a recombination hotspot. While the highly variable *porA*, *porB*, *fetA*, *nhba* and *opcA* loci showed no variation, the C1, C2 and C3 strains harboured a different *fhbP* allele from the C4 and C5 strains. The *fhbP* gene encodes factor H-binding protein, a key virulence factor and vaccine antigen. More than half of the genes affected by the 17 identified HGT events encoded proteins of the ‘cell envelope’ and the ‘transport/binding protein’ categories. The accumulation of changes in cell-surface structures may be the result of selection imposed by natural immunity developing in the local host population. Genomic epidemiology studies may therefore represent an eminent approach for identifying targets of natural immune responses, with major implications for the design of next-generation protein-based subunit vaccines. Furthermore, improvement of our understanding of meningococcal population dynamics, and the basis of meningococcal virulence, will lead to the development of better vaccines and enhance our ability to recognize outbreaks and epidemics earlier, which in turn may lead to earlier and more appropriate interventions.

## Data bibliography

1. Complete DNA sequence of a serogroup A strain of *Neisseria meningitidis* Z2491. Genbank accession number AL157959.1 (2000).
2. Whole-genome sequence of the transformable *Neisseria meningitidis* serogroup A strain WUE2594. Genbank accession number FR774048.1 (2011).
3. Emergence of a new epidemic *Neisseria meningitidis* serogroup A Clone in the African meningitis belt: high-resolution picture of genomic changes that mediate immune evasion. ENA accession number ERS041026, ERS040961 (2016).
4. Systems that modulate homologous recombination. Genbank accession numbers CP002422, CP002421, CP002423, CP002420.1, CP002424, CP002419.1 (2011).
5. Comparative genome biology of a serogroup B carriage and disease strain supports a polygenic nature of meningococcal virulence. Genbank accession number CP001561.1 (2010).
6. Complete genome sequence of *Neisseria meningitidis* serogroup B strain MC58. Genbank accession number AE002098.2 (2000).
7. Meningococcal genetic variation mechanisms viewed through comparative analysis of serogroup C strain FAM18. Genbank accession number AM421808.1 (2007).
8. NeMeSys: a biological resource for narrowing the gap between sequence and function in the human pathogen *Neisseria meningitidis*. Genbank accession number FM999788.1 (2009).
9. The Complete Genome Sequence of *Neisseria gonorrhoeae*. Genbank accession number AE004969.1 (2013).
10. Draft Genome Sequence of a Dominant, Multidrug-Resistant *Neisseria gonorrhoea* Strain, TCDC-NG08107, from a Sexual Group at High Risk of Acquiring Human Immunodeficiency Virus Infection and Syphilis. Genbank accession number# PRJNA61377 (2011)
11. Independent evolution of the core and accessory gene sets in the genus *Neisseria*: insights gained from the genome of *Neisseria lactamica* isolate 020-06. Genbank accession numberFN995097.1 (2010)

## Supplementary Data

2672 Supplementary File 1
