# Supplementary material for: Emergence and genomic diversification of a virulent serogroup W:ST-2881(CC175) Neisseria meningitidis clone in the African meningitis belt
Source: Microb Genom. 2017 Jun 21;3(8):e000120. doi: 10.1099/mgen.0.000120 (PMC5610715; doi:10.1099/mgen.0.000120)
Supplement: Supplementary File 1 [file mgen-3-120-s001.pdf]

Supplementary Table 1: Mapping statistics and metadata for the bacterial isolates used in the study.

Isolate	Accession Number	Serogroup	Year of isolation	Origin	Source	ST	Coverage	% mapped
2039	ERR369484	W	2005	NDSS, Burkina Faso	CSF	ST-2881	375,529	86,17
2363	ERR369485	W	2006	NDSS, Burkina Faso	pharynx	ST-2881	357,374	85,38
2400	ERR369486	W	2006	IRA, Burkina Faso	pharynx	ST-2881	351,697	85,86
2841	ERR332366	W	2008	IRA, Burkina Faso	pharynx	ST-2881	270,718	85,1
2855	ERR369488	W	2008	IRA, Burkina Faso	pharynx	ST-2881	417,958	85,78
2863	ERR332367	W	2008	IRA, Burkina Faso	pharynx	ST-2881	236,628	84,95
2882	ERR332368	W	2009	NDSS, Burkina Faso	pharynx	ST-2881	283,032	84,78
2959	ERR369489	W	2009	IRA, Burkina Faso	pharynx	ST-2881	392,928	86,14
2973	ERR332369	W	2009	IRA, Burkina Faso	pharynx	ST-2881	241,986	84,87
2976	ERR349880	W	2010	KND, Ghana	CSF	ST-2881	224,872	85,38
2978	ERR332305	W	2010	KND, Ghana	CSF	ST-2881	292,728	84,53
2980	ERR332306	W	2010	KND, Ghana	CSF	ST-2881	263,148	84,32
2998	ERR332307	W	2010	KND, Ghana	CSF	ST-2881	285,969	84,61
3000	ERR332317	W	2010	KND, Ghana	CSF	ST-2881	268,41	84,75
3001	ERR332308	W	2010	KND, Ghana	CSF	ST-2881	249,919	84,59
3002	ERR332309	W	2010	KND, Ghana	CSF	ST-2881	273,202	84,71
3007	ERR332318	W	2010	KND, Ghana	CSF	ST-2881	323,401	84,68
3009	ERR332310	W	2010	KND, Ghana	CSF	ST-2881	354,519	84,77
3013	ERR332319	W	2010	KND, Ghana	CSF	ST-2881	292,58	84,77
3017	ERR332311	W	2010	KND, Ghana	CSF	ST-2881	275,021	84,93
3048	ERR332312	W	2010	KND, Ghana	CSF	ST-2881	287,531	84,23
3049	ERR332313	W	2010	KND, Ghana	CSF	ST-2881	256,389	84,7
3050	ERR369490	W	2010	KND, Ghana	CSF	ST-2881	382,038	85,75
3051	ERR332314	W	2010	KND, Ghana	CSF	ST-2881	314,794	84,46
3052	ERR369491	W	2010	KND, Ghana	CSF	ST-2881	392,177	85,86
3053	ERR332315	W	2010	KND, Ghana	CSF	ST-2881	283,889	84,49

3055	ERR369492	W	2010	KND, Ghana	CSF	ST-2881	387,458	85,77
3056	ERR369493	W	2010	KND, Ghana	CSF	ST-2881	321,422	85,84
3059	ERR332316	W	2010	KND, Ghana	CSF	ST-2881	279,14	84,34
3086	ERR369494	W	2010	KND, Ghana	CSF	ST-2881	365,267	85,86
3089	ERR369495	W	2010	KND, Ghana	CSF	ST-2881	363,712	85,9
3090	ERR369496	W	2010	KND, Ghana	CSF	ST-2881	364,878	85,52
3092	ERR369498	W	2010	KND, Ghana	CSF	ST-2881	402,807	85,7
3095	ERR369499	W	2010	KND, Ghana	CSF	ST-2881	381,938	85,65
3137	ERR332339	W	2009	KND, Ghana	pharynx	ST-2881	275,695	84,61
3144	ERR369500	W	2009	KND, Ghana	pharynx	ST-2881	387,285	85,19
3146	ERR332321	W	2009	KND, Ghana	pharynx	ST-2881	264,139	84,47
3151	ERR332322	W	2009	KND, Ghana	pharynx	ST-2881	294,747	85,01
3152	ERR332323	W	2009	KND, Ghana	pharynx	ST-2881	270,913	84,55
3155	ERR369501	W	2009	KND, Ghana	pharynx	ST-2881	346,547	85,96
3161	ERR332320	W	2009	KND, Ghana	pharynx	ST-2881	286,409	84,9
3163	ERR369502	W	2009	KND, Ghana	pharynx	ST-2881	358,432	85,73
3164	ERR332324	W	2009	KND, Ghana	pharynx	ST-2881	262,444	84,49
3165	ERR369503	W	2009	KND, Ghana	pharynx	ST-2881	356,598	85,8
3169	ERR369504	W	2009	KND, Ghana	pharynx	ST-2881	393,325	85,88
3170	ERR369505	W	2009	KND, Ghana	pharynx	ST-2881	403,866	85,84
3185	ERR369506	W	2010	KND, Ghana	pharynx	ST-2881	393,632	85,93
3187	ERR369507	W	2010	KND, Ghana	pharynx	ST-2881	377,105	85,58
3189	ERR349881	W	2010	KND, Ghana	pharynx	ST-2881	239,062	84,38
3190	ERR369508	W	2010	KND, Ghana	pharynx	ST-2881	402,935	85,79
3191	ERR369509	W	2010	KND, Ghana	pharynx	ST-2881	372,764	85,8
3196	ERR332340	W	2010	KND, Ghana	pharynx	ST-2881	254,516	84,64
3197	ERR369510	W	2010	KND, Ghana	pharynx	ST-2881	314,021	85,92
3198	ERR369511	W	2010	KND, Ghana	pharynx	ST-2881	385,057	85,87

3199	ERR369512	W	2010	KND, Ghana	pharynx	ST-2881	357,036	85,78
3200	ERR369513	W	2010	KND, Ghana	pharynx	ST-2881	371,491	85,55
3218	ERR332325	W	2010	KND, Ghana	pharynx	ST-2881	279,301	84,75
3219	ERR369514	W	2010	KND, Ghana	pharynx	ST-2881	404,071	85,77
3220	ERR332326	W	2010	KND, Ghana	pharynx	ST-2881	339,342	84,77
3222	ERR332327	W	2010	KND, Ghana	pharynx	ST-2881	271,049	84,84
3223	ERR332328	W	2010	KND, Ghana	pharynx	ST-2881	299,123	84,48
3224	ERR332329	W	2010	KND, Ghana	pharynx	ST-2881	288,426	84,72
3250	ERR332330	W	2010	KND, Ghana	pharynx	ST-2881	290,762	84,57
3253	ERR332335	W	2010	KND, Ghana	pharynx	ST-2881	306,288	84,39
3255	ERR332331	W	2010	KND, Ghana	pharynx	ST-2881	282,937	84,7
3257	ERR332336	W	2010	KND, Ghana	pharynx	ST-2881	309,331	84,49
3261	ERR332332	W	2010	KND, Ghana	pharynx	ST-2881	249,397	84,49
3268	ERR332333	W	2010	KND, Ghana	pharynx	ST-2881	247,176	84,49
3270	ERR332337	W	2010	KND, Ghana	pharynx	ST-2881	275,59	84,79
3273	ERR369516	W	2010	KND, Ghana	pharynx	ST-2881	394,954	85,92
3274	ERR332334	W	2010	KND, Ghana	pharynx	ST-2881	368,436	85,03
3275	ERR369517	W	2010	KND, Ghana	pharynx	ST-2881	339,818	85,86
3326	ERR369522	W	2010	KND, Ghana	pharynx	ST-2881	423,7	85,66
3327	ERR369523	W	2010	KND, Ghana	pharynx	ST-2881	371,496	85,89

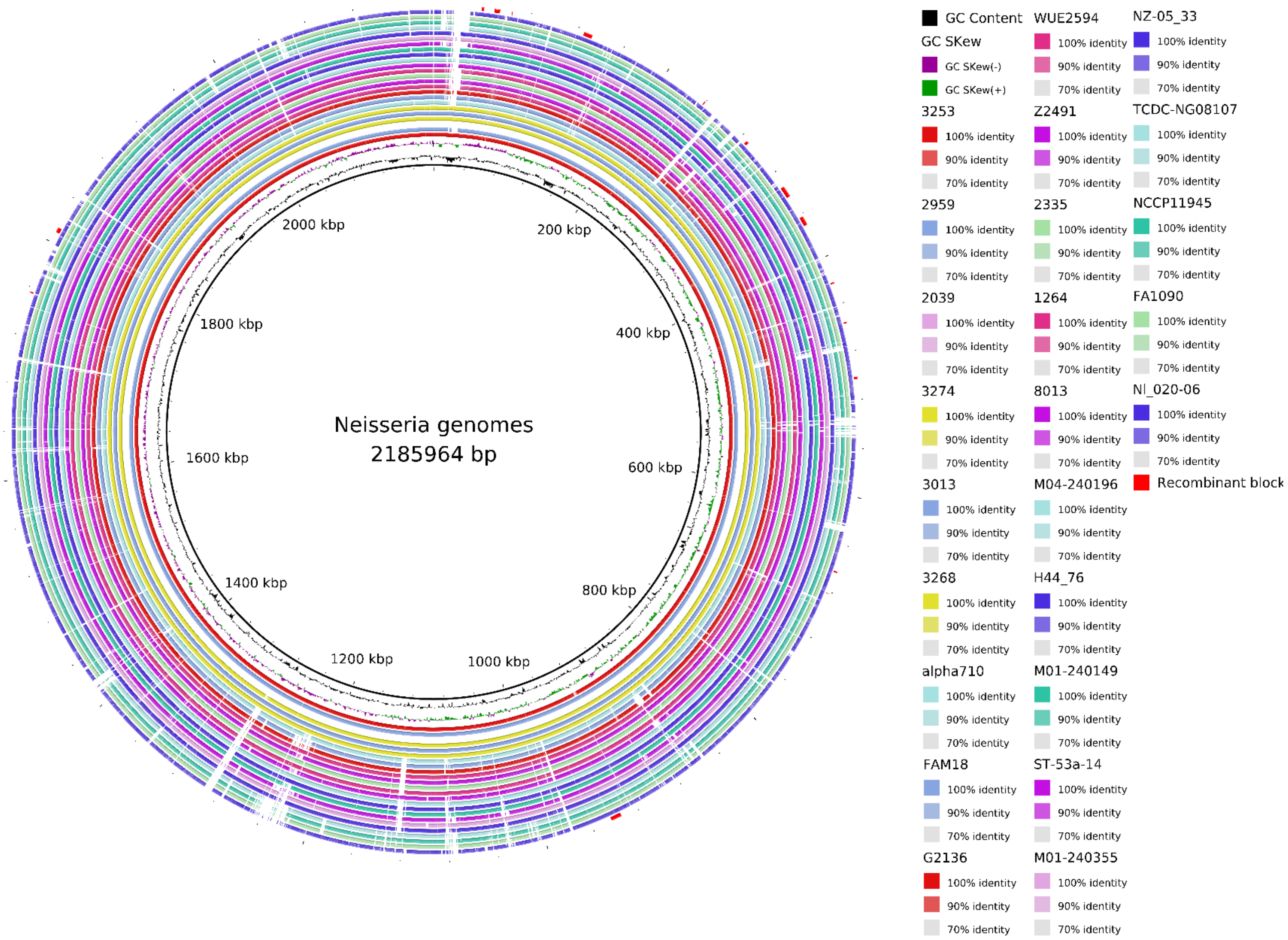

Supplementary Figure 1:

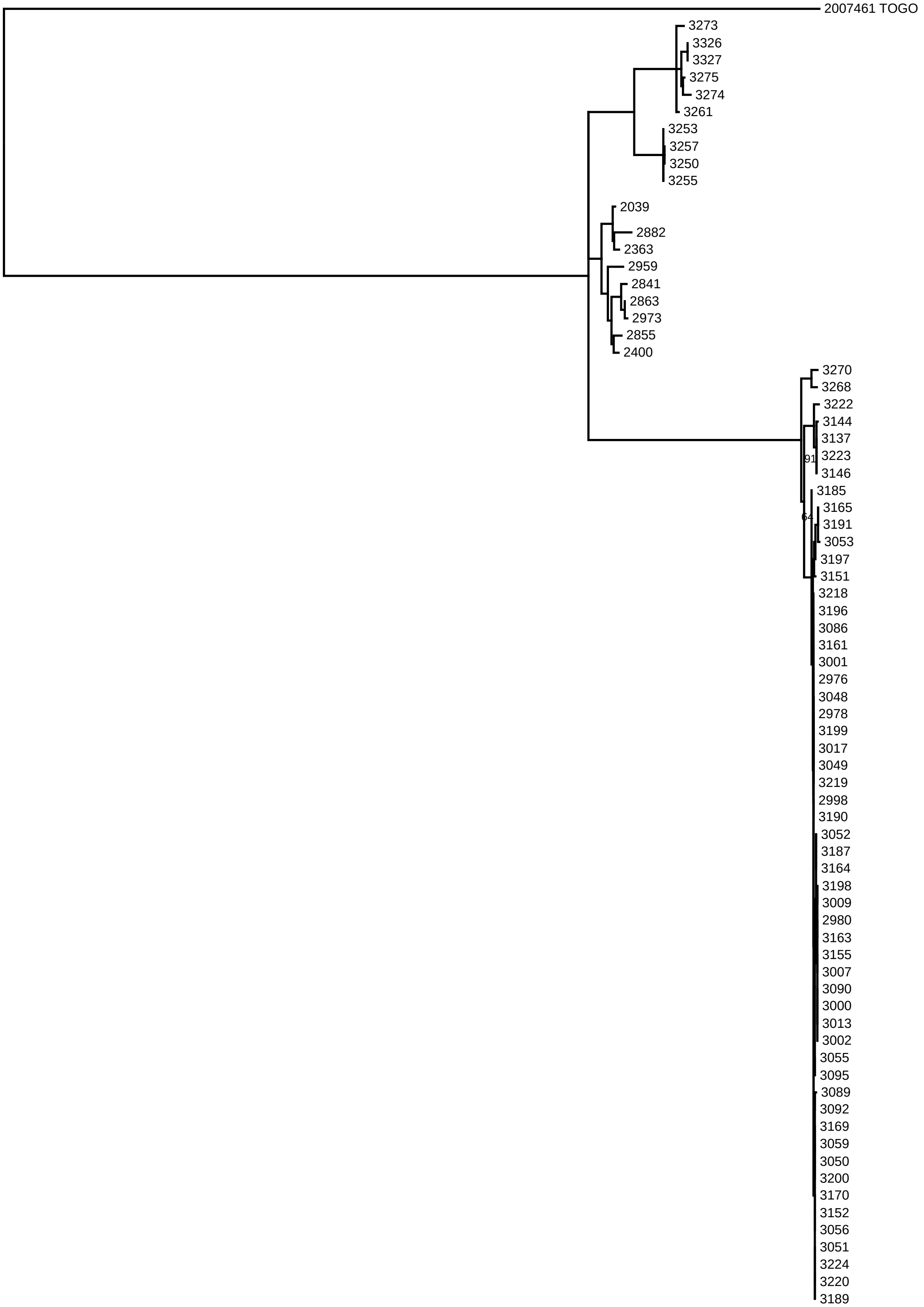

Supplementary Figure 2:

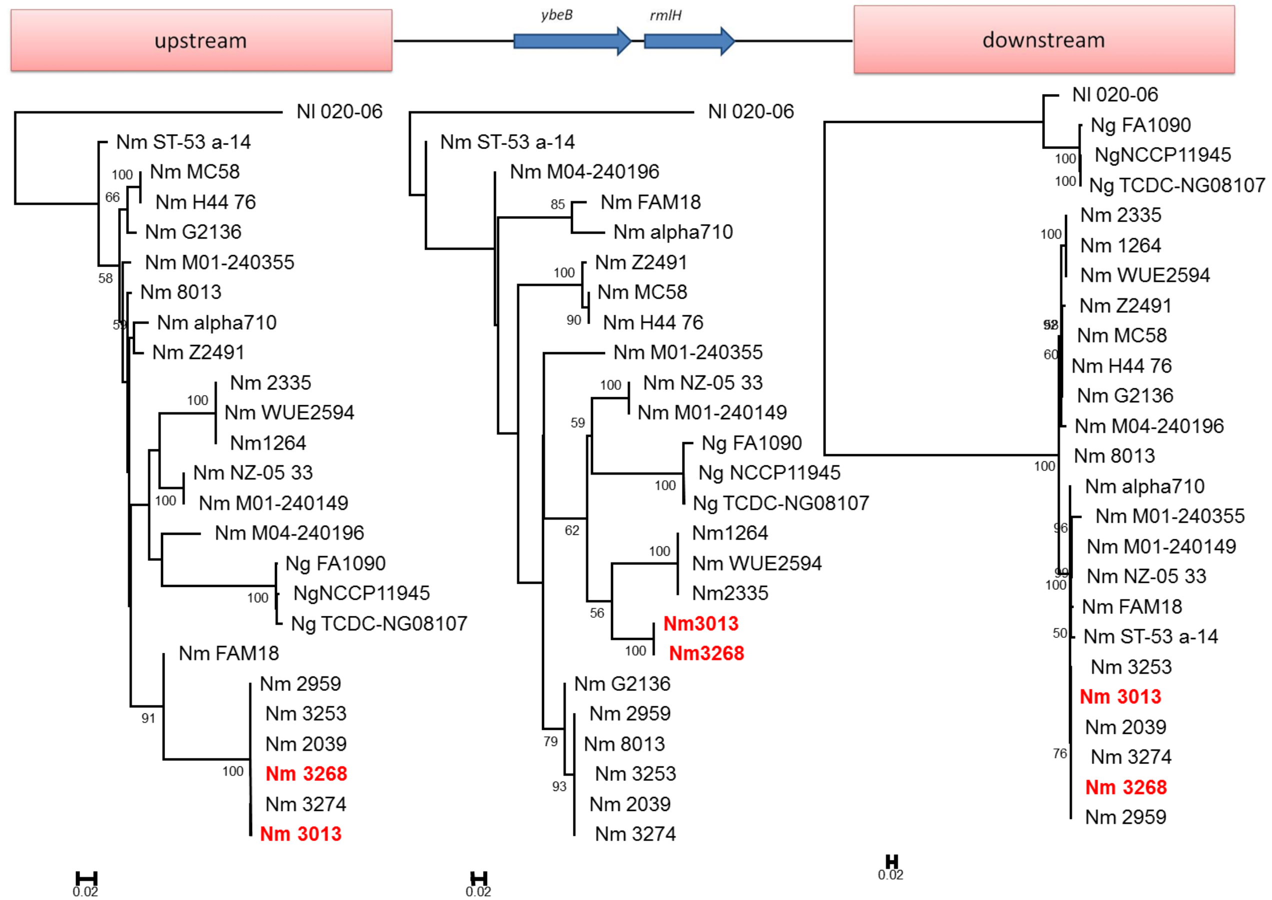

Id_gene	Name	Product	Id_gene	Name	Product
NM2039_00241	*ybeB*	hypothetical protein	NM2039_00242	*rmlH*	rRNA large subunit methyltransferase

Supplementary Figure 3:

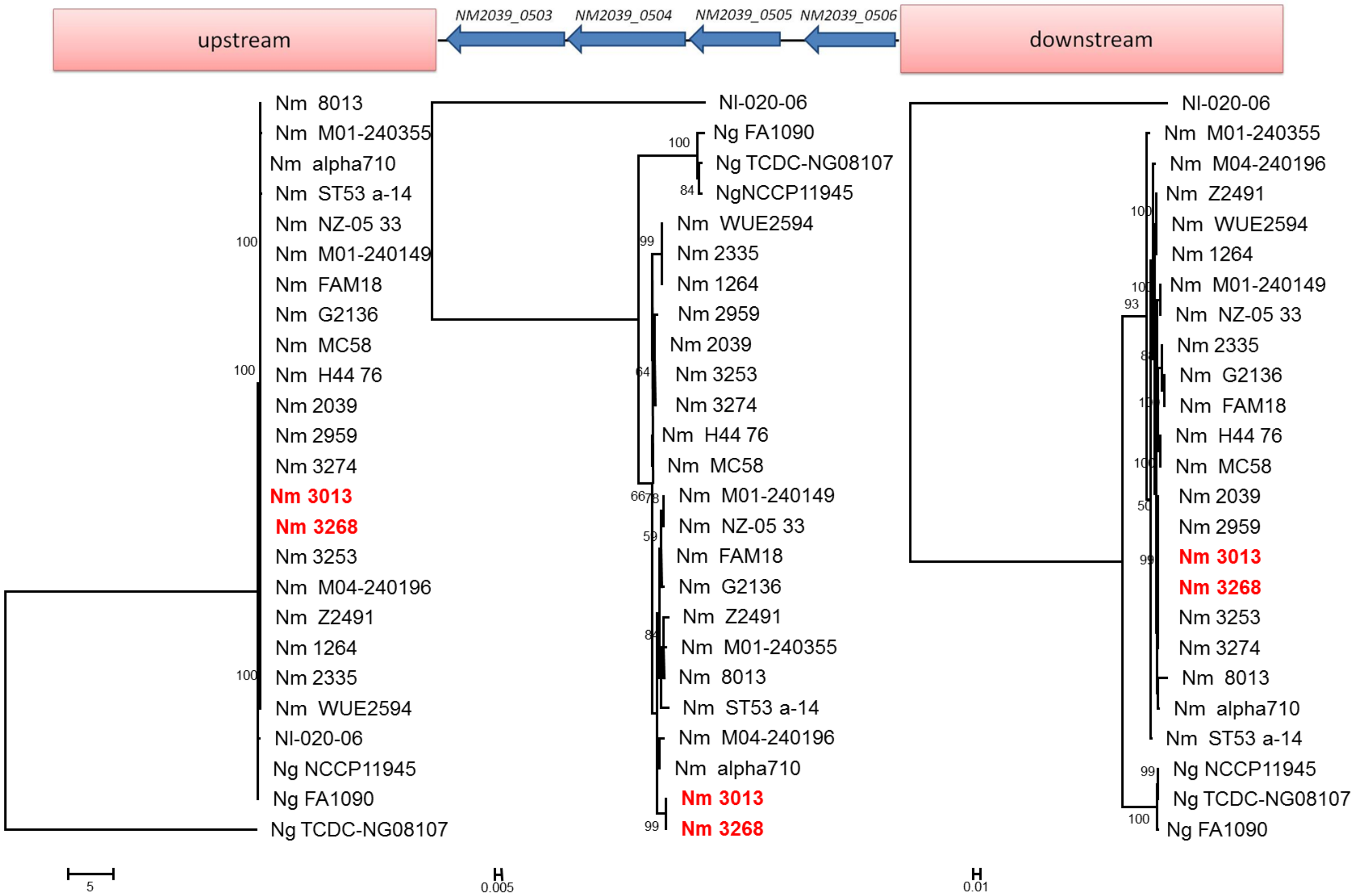

**Id_gene**	**Name**	**Product**	**Id_gene**	**Name**	**Product**
NM2039_00503	*pilP*	PilP protein	NM2039_00505	*pilN*	Fimbrial assembly protein (PilN)
NM2039_00504	*pilO*	Pilus assembly protein, PilO	NM2039_00506	*pilM*	Type IV pilus assembly protein PilM

Supplementary Figure 4:

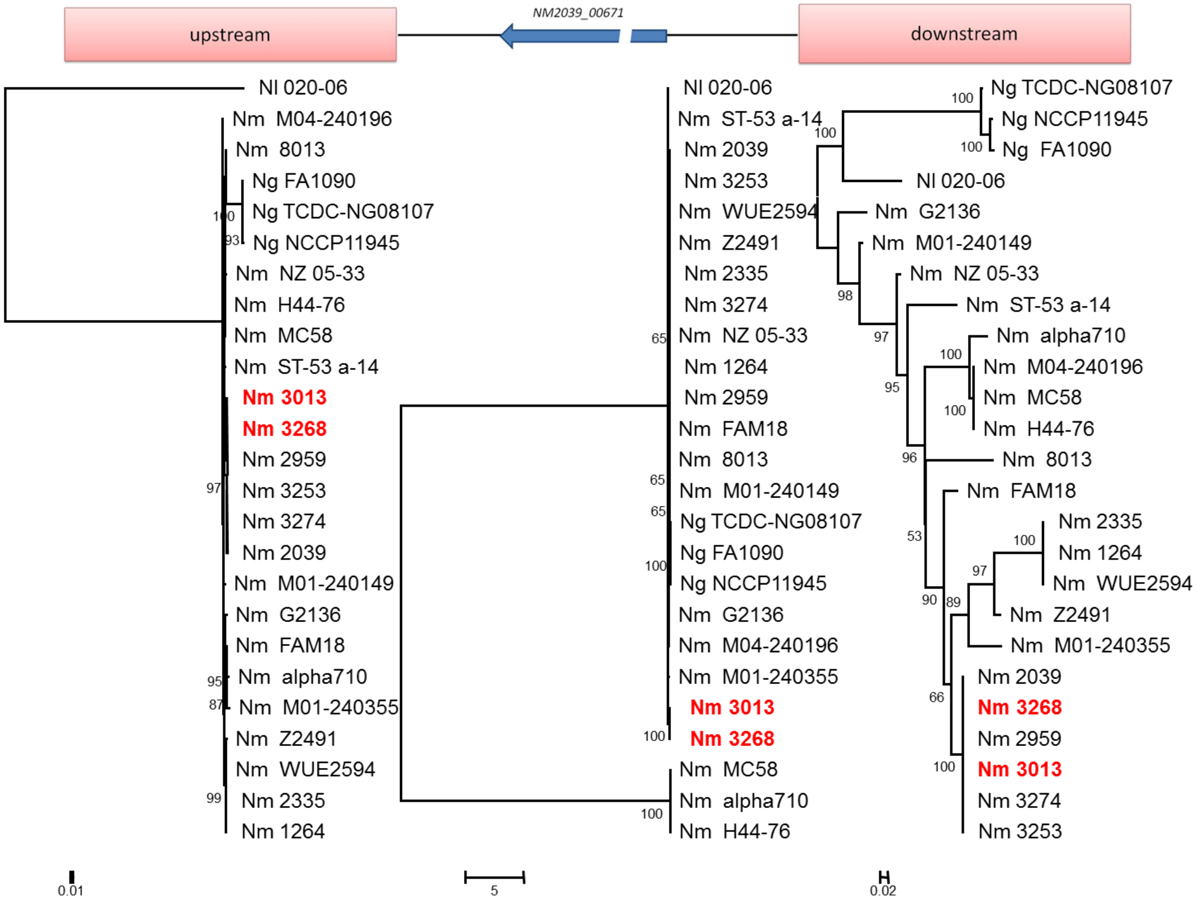

Id_gene	Name	Product
NM2039_00671		hypothetical protein

Supplementary Figure 5:

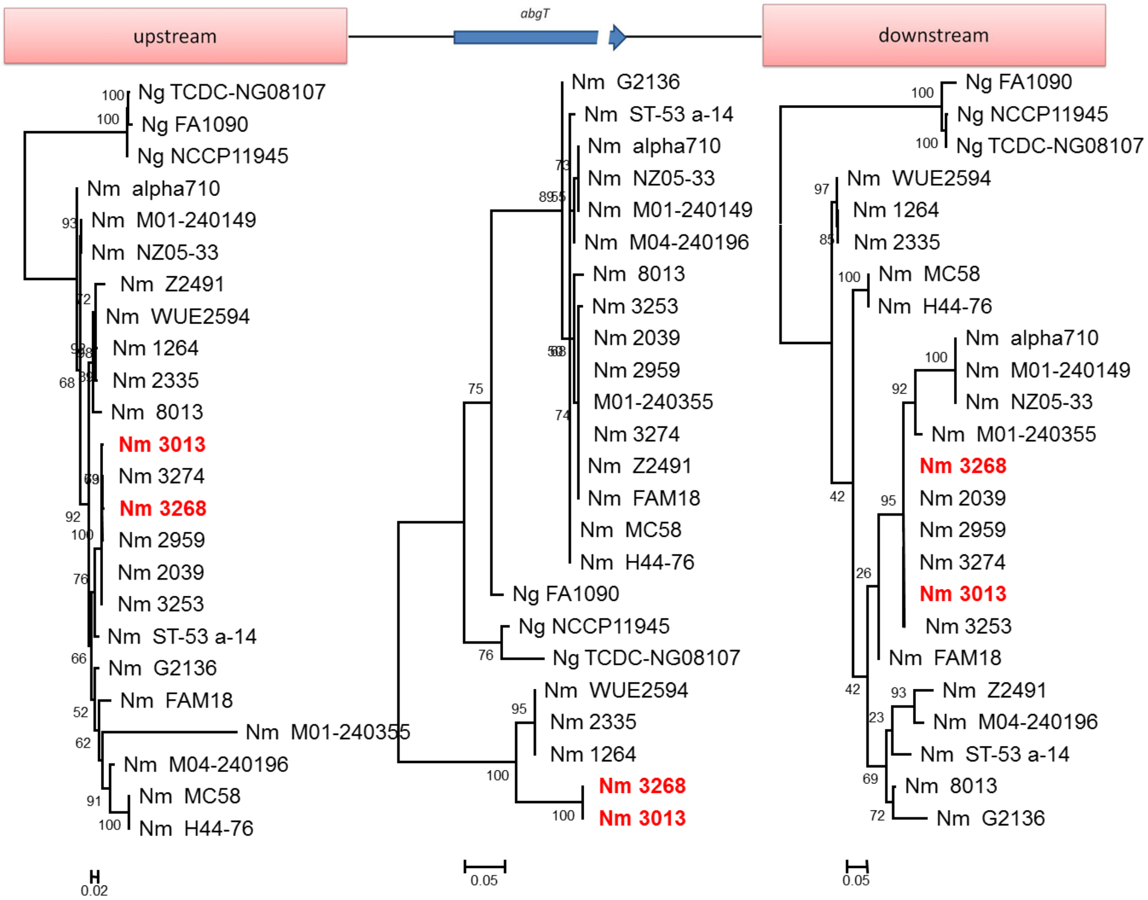

Id_gene	Name	Product
NM2039_01723	*abgT*	antibiotic resistance efflux pump component

Supplementary Figure 6:

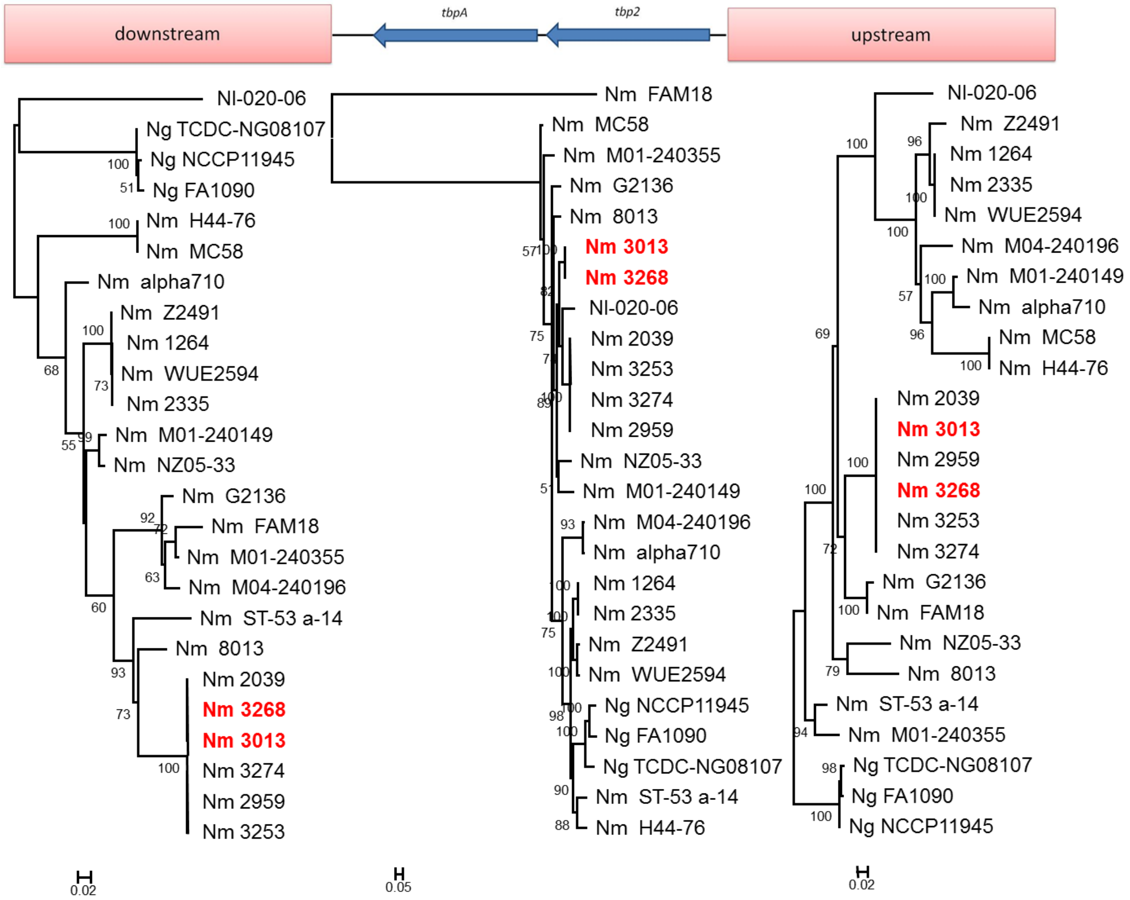

Id_gene	Name	Product	Id_gene	Name	Product
NM2039_01774	*tbpA*	transferrin-binding protein 1	NM2039_01775	*Tbp2*	transferrin-binding protein B

Supplementary Figure 7:

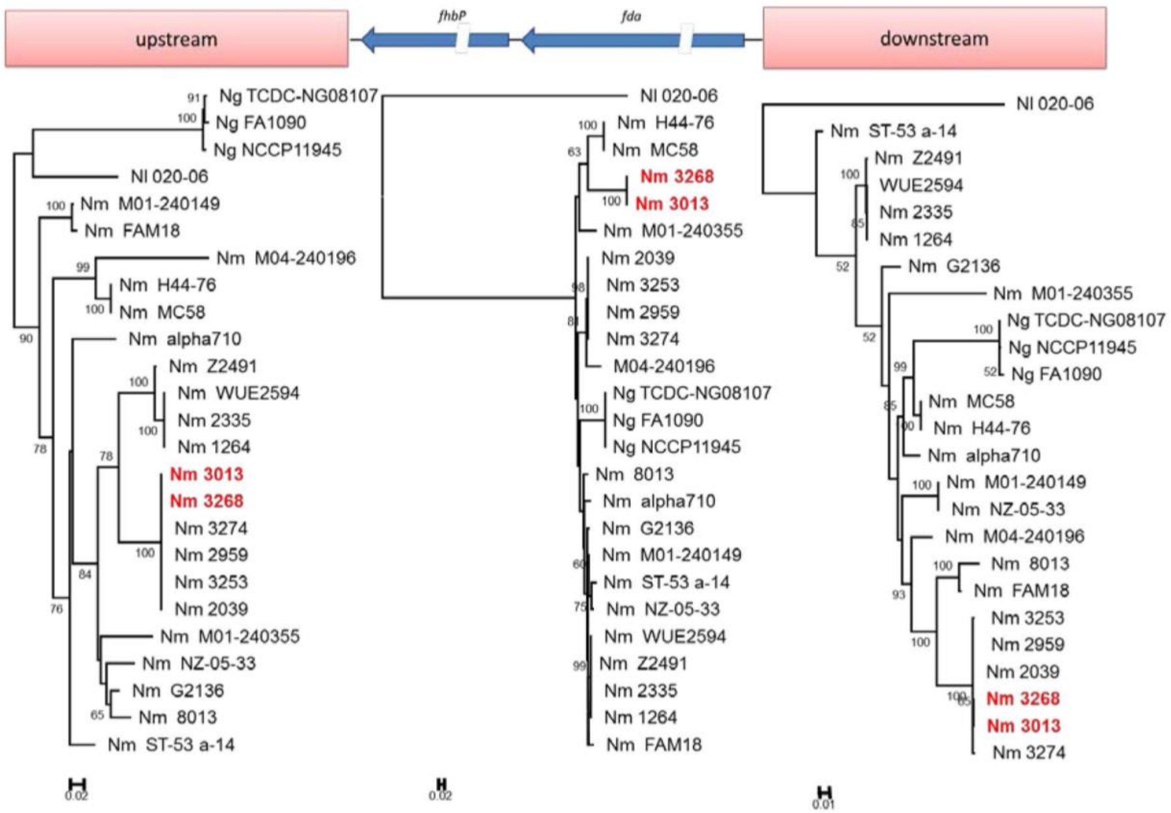

Id_gene	Name	Product	Id_gene	Name	Product
NM2039_00413	*fda*	fructose-1,6-bisphosphate aldolase	NM2039_00412	fhbP	Factor H-binding protein

Supplementary Figure 8:

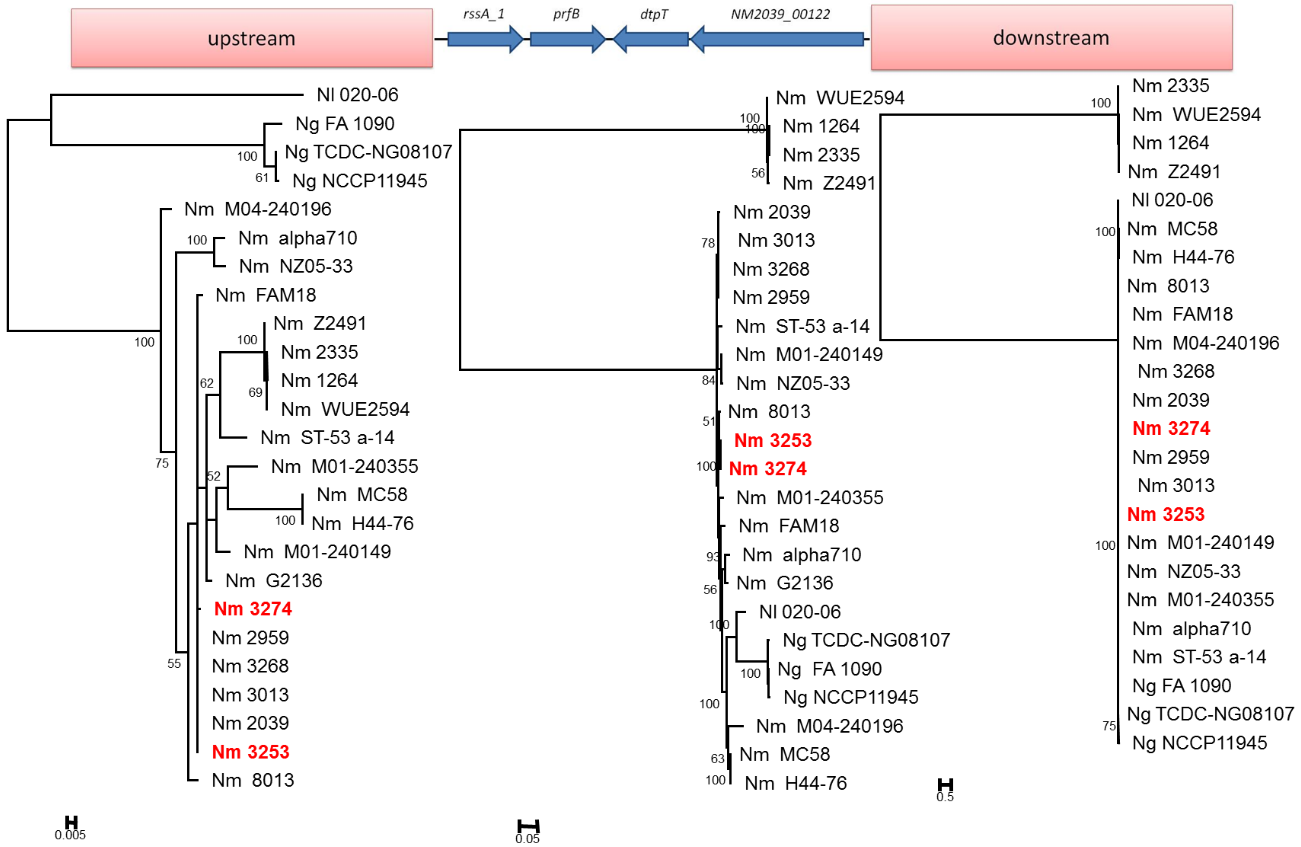

Id_gene	Name	Product
NM2039_00117	*rssA1*	Possible lipoprotein,NTE family protein rssA,hypothetical protein,Patatin-like phospholipase
NM2039_00118	*prfB*	peptide chain release factor 2
NM2039_00121	*dtpT*	peptide transporter ,Di-/tripeptide transporter,putative tripeptide transporter permease,amino acid/peptide transporter
NM2039_00122		Periplasmic protein,hypothetical protein,Family of unknown function (DUF490)

Supplementary Figure 9:

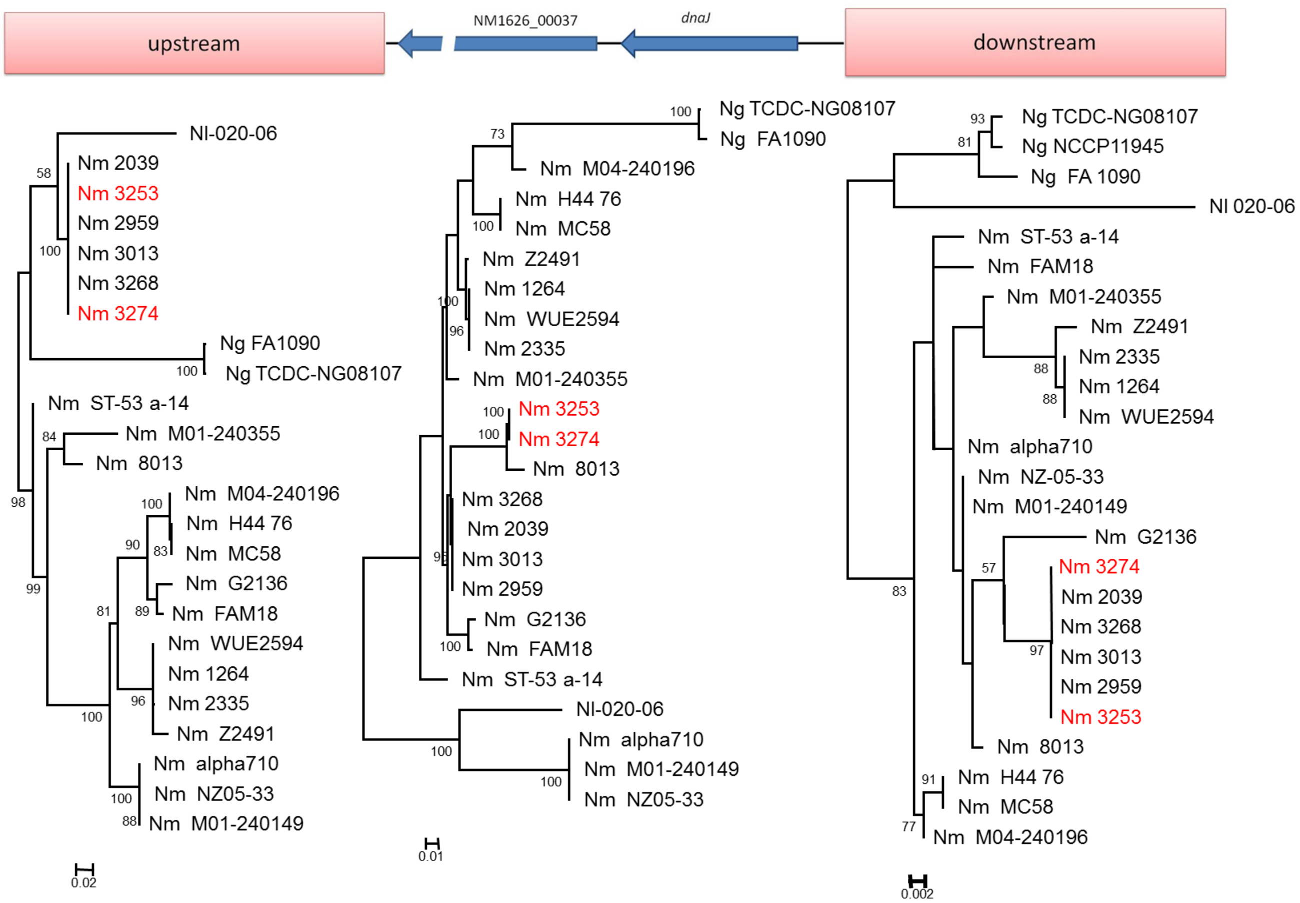

Id_gene	Name	Product	Id_gene	Name	Product
NM2039_00037		membrane protein	NM2039_00038	DnaJ_1	chaperone protein DnaJ

Supplementary Figure 10:

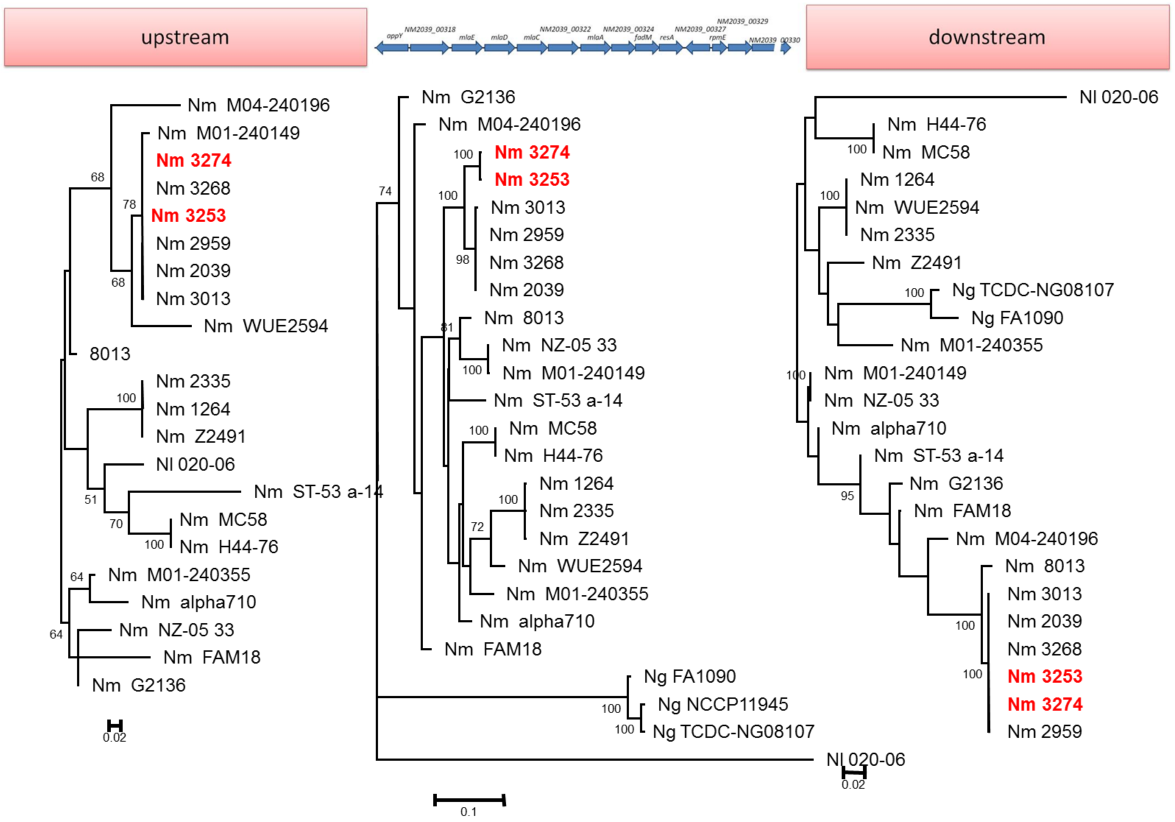

Id_gene	Name	Product	Id_gene	Name	Product
NM2039_00317	*appY*	transcriptional regulator	NM2039_00324		VacJ-like protein
NM2039_00318		ABC transporter ATP-binding protein	NM2039_00325		hioesterase family protein
NM2039_00319	*mlaE*	ABC transporter inner membrane protein	NM2039_00326		putative thioredoxin
NM2039_00320	*mlaD*	outer membrane transport protein	NM2039_00327		acetyltransferase
NM2039_00321	*mlaC*	Periplasmic transport protein	NM2039_00328	*rpmE*	50S ribosomal protein L31
NM2039_00322		NTP binding protein	NM2039_00329		cadmium resistance protein
NM2039_00323	*mlaA*	putative VacJ-like protein	NM2039_00330		inner membrane protein

Supplementary Figure 11:

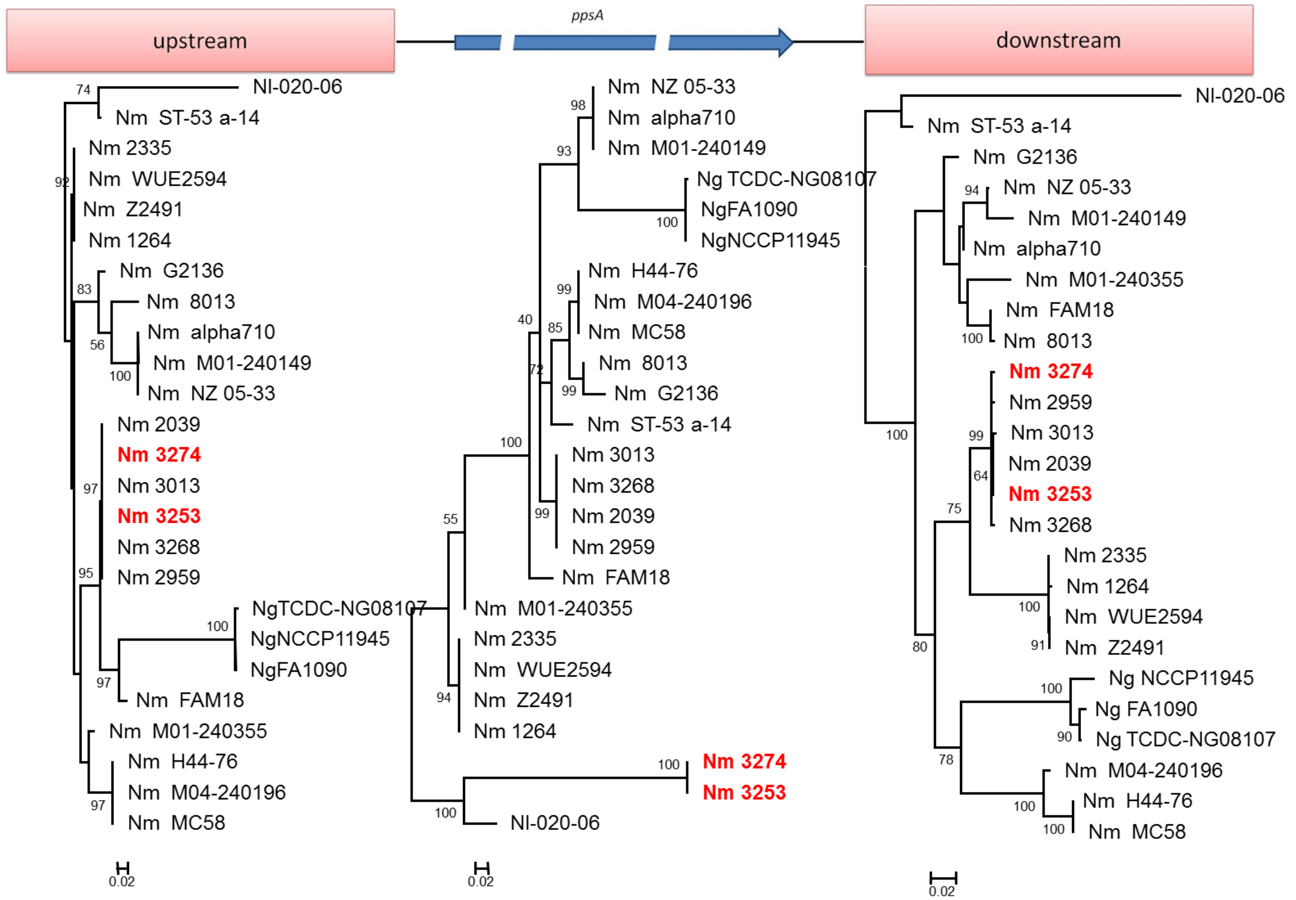

Id_gene	Name	Product
NM2039_00653	*ppsA*	phosphoenolpyruvate synthase

Supplementary Figure 12:

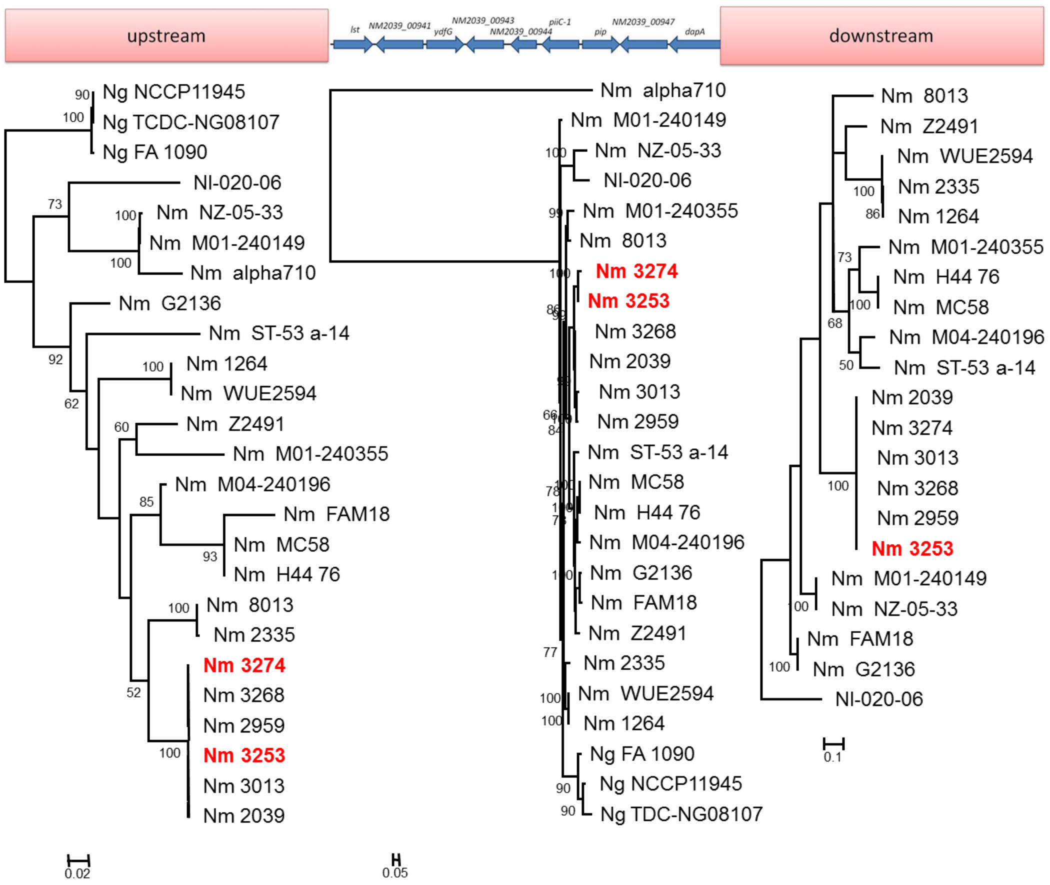

Id_gene	Name	Product	Id_gene	Name	Product
NM2039_00940	*lst*	CMP-N-acetylneuraminate-beta-galactosamide- alpha-2,3-sialyltransferase	NM2039_00945	*piiC_1*	opacity protein
NM2039_00941		C-type cytochrome	NM2039_00946	*pip*	proline iminopeptidase
NM2039_00942	*ydfG*	oxidoreductase,NADP-dependent 3-hydroxy acid dehydrogenase YdfG	NM2039_00947		lipoprotein
NM2039_00943		acyl-CoA hydrolase	dapA		dihydrodipicolinate synthase
NM2039_00944		hypothetical protein			

Supplementary Figure 13:

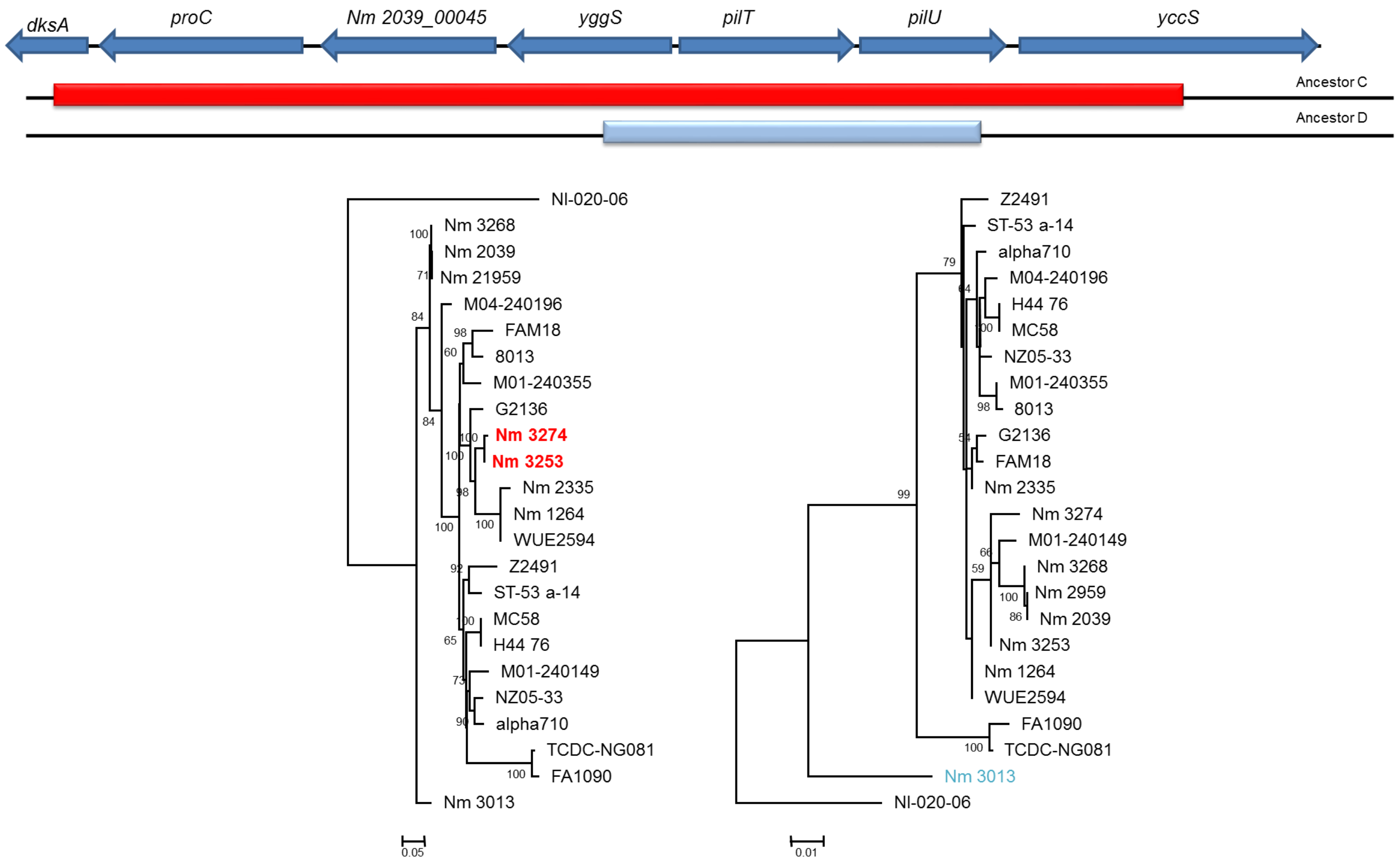

Id_gene	Name	Product	Id_gene	Name	Product
NM2039_00043	*dksA*	DskA protein	NM2039_00047	*pilT*	twitching motility/pilus retraction protein
NM2039_00044	*proC*	pyrroline-5-carboxylate reductase	NM2039_00048	*pilU*	witching motility - like protein
NM2039_00045		lipoprotein	NM2039_00049	*yccS*	integral membrane protein
NM2039_00046	*yggS*	hypothetical protein			

Supplementary Figure 14:

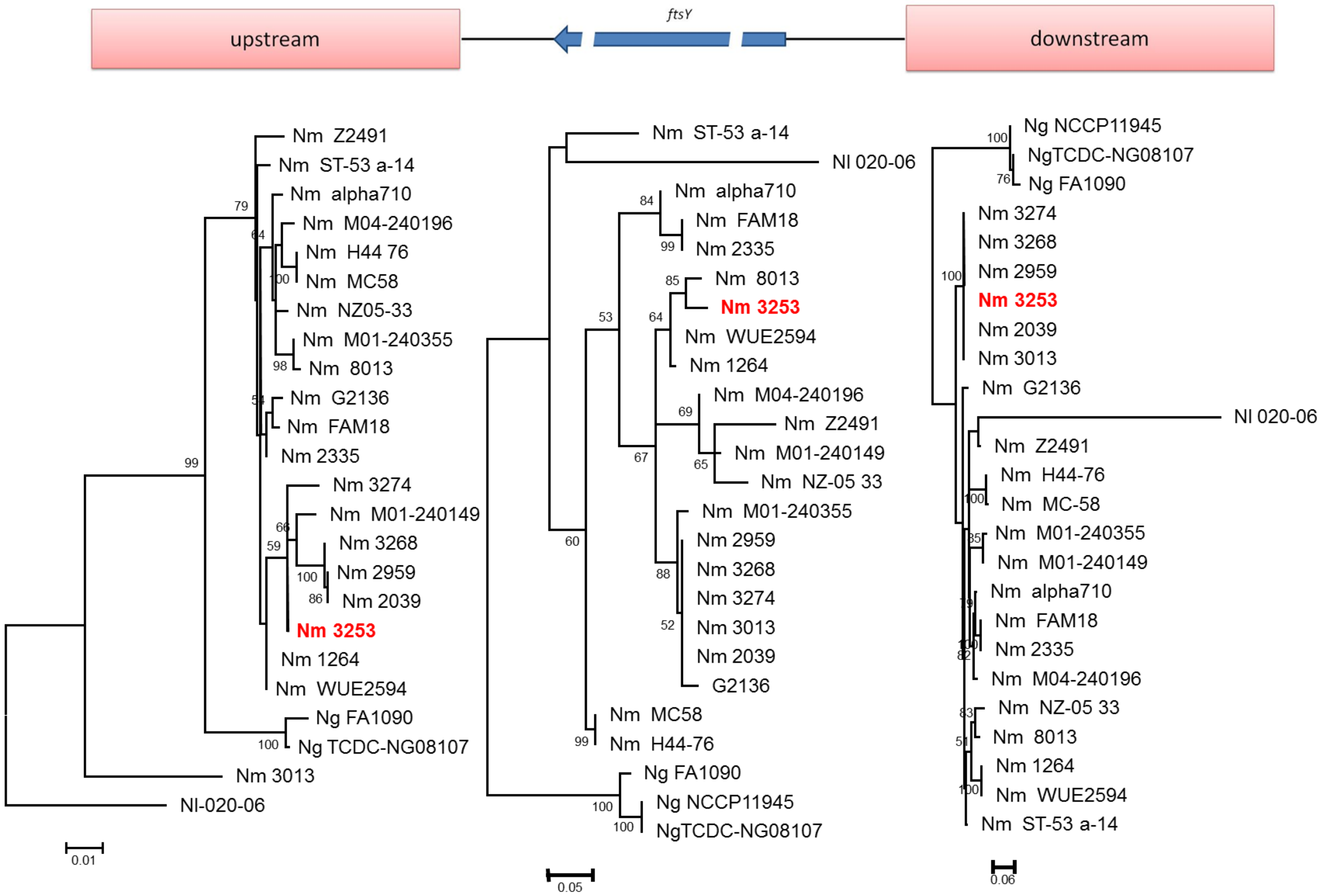

Id_gene	Name	Product
NM2039_00052	*ftsY*	signal recognition particle protein

Supplementary Figure 15:

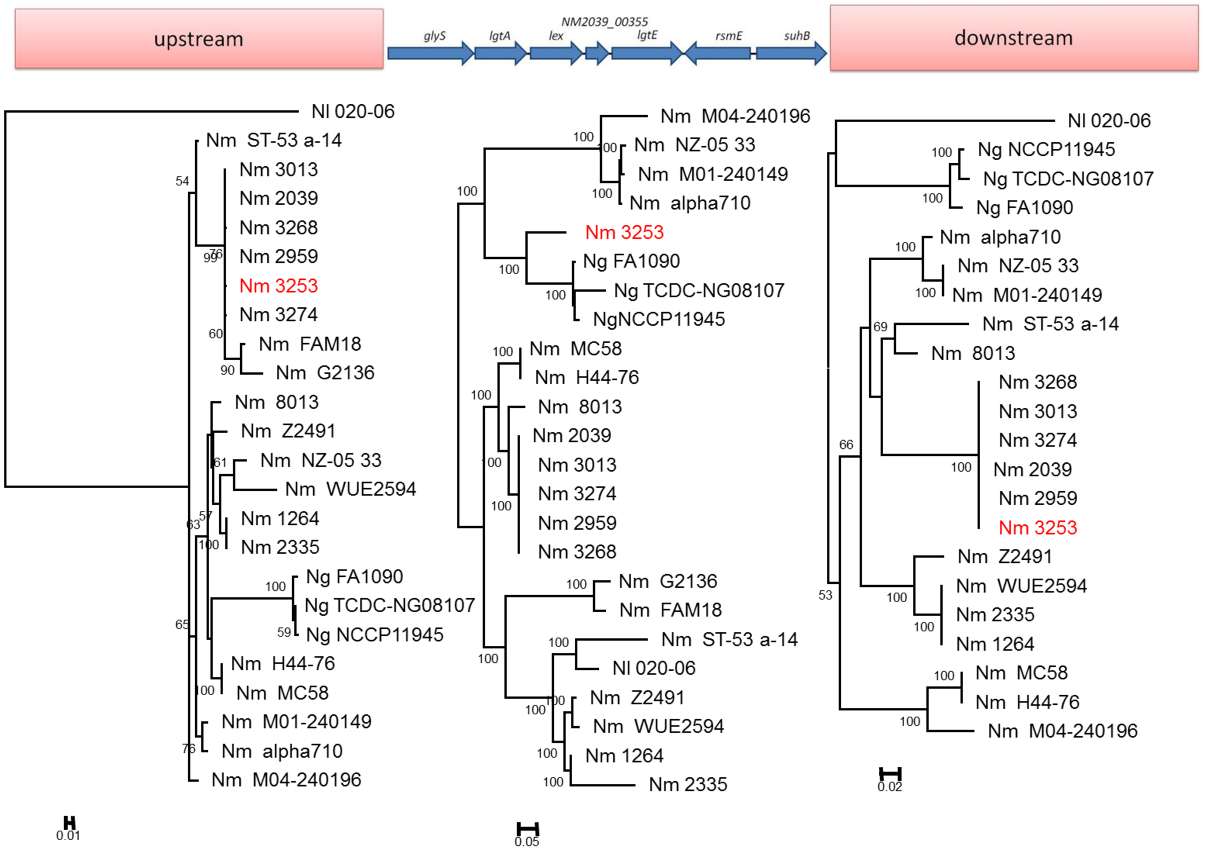

Id_gene	Name	Product	Id_gene	Name	Product
NM2039_00352	*glyS*	glycyl-tRNA synthetase subunit beta	NM2039_00356	*lgtE*	lacto-N-neotetraose biosynthesis glycosyl transferase LgtE
NM2039_00353	*lgtA*	lacto-N-neotetraose biosynthesis glycosyl transferase	NM2039_00357	*rsmE*	16S ribosomal RNA methyltransferase RsmE
NM2039_00354	*Lex1*	lacto-N-neotetraose biosynthesis glycosyl tranferase	NM2039_00358	*suhB_1*	Myo-inositol-1(Or 4)-monophosphatase
NM2039_00355		lacto-N-neotetraose biosynthesis glycosyl transferase-like protein			

Supplementary Figure 16:

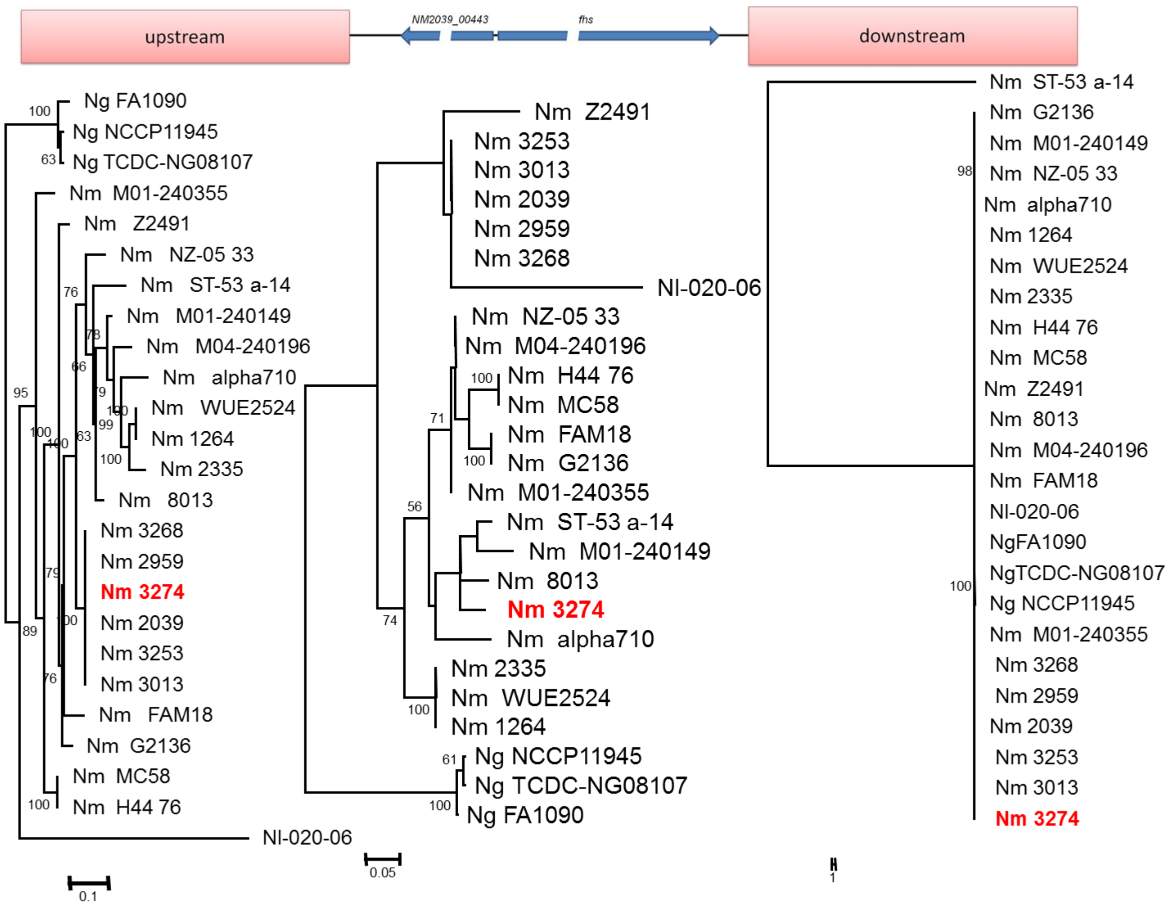

Id_gene	Name	Product	Id_gene	Name	Product
NM2039_00443		integral membrane protein	NM2039_00444	*fhs*	formate--tetrahydrofolate ligase

Supplementary Figure 17:

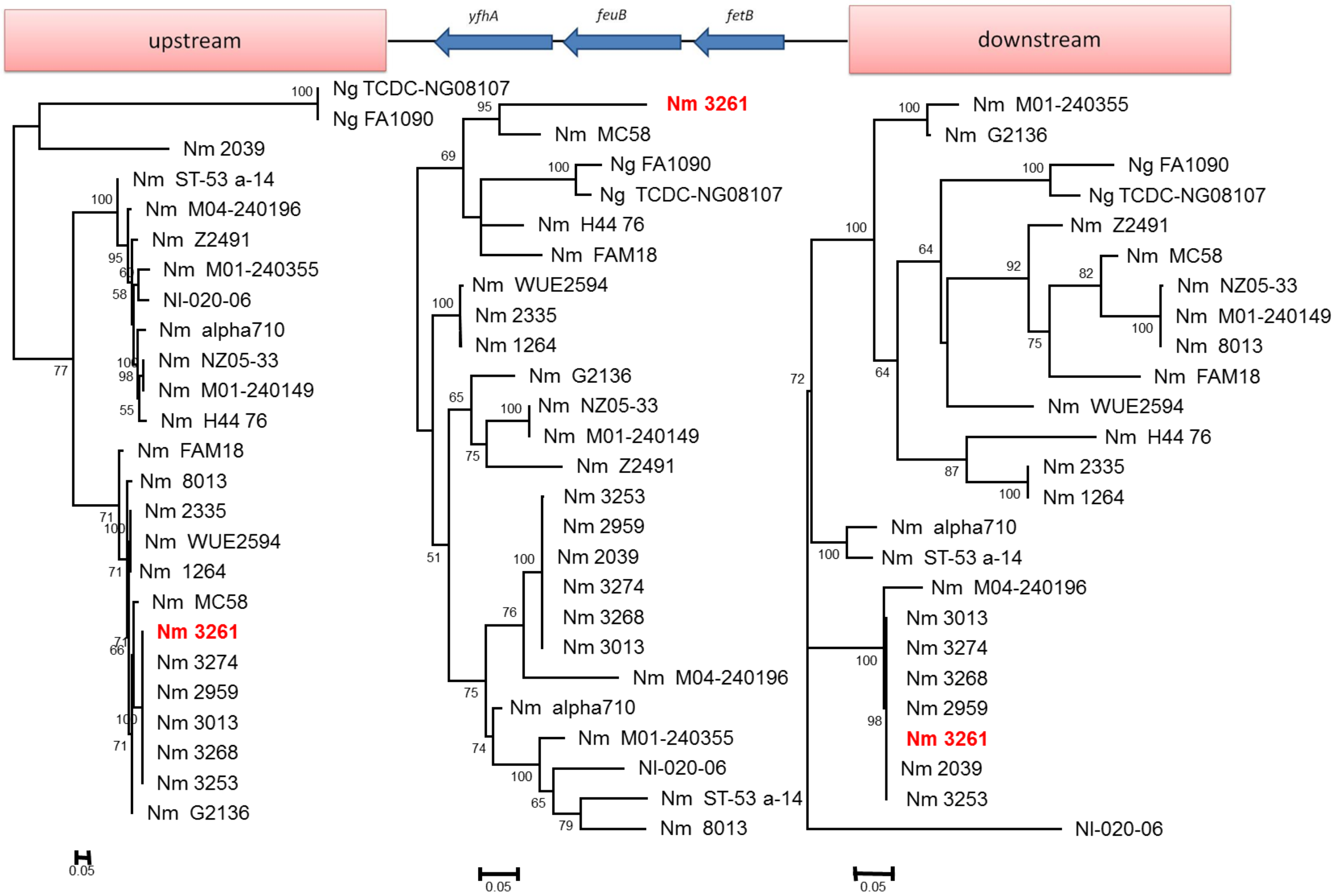

Id_gene	Name	Product	Id_gene	Name	Product
NM2039_00284	*yfhA*	ABC transporter permease	NM2039_00286	*fetB*	membrane transport solute-binding protein
NM2039_00285	*feuB*	ABC transporter permease			

Supplementary Figure 18:
